# Stability-aware LSTM–MPC framework for personalized levothyroxine dosing in Hashimoto’s thyroiditis

**DOI:** 10.3389/fendo.2026.1894583

**Published:** 2026-08-13

**Authors:** Ravi Sharma, Uwe Riedmann, Stefan Pilz, Markus Reichhartinger

**Affiliations:** 1 Institute of Automation and Control, Graz University of Technology, Graz, Austria; 2 Division of Endocrinology and Diabetology, Department of Internal Medicine, Medical University of Graz, Graz, Austria

**Keywords:** Hashimoto’s thyroiditis, levothyroxin treatment, LSTM (Long Short-Term Memory Networks), model predictive control (MPC), stability analysis, thyroid treatment

## Abstract

Hypothyroidism, most commonly caused by Hashimoto’s thyroiditis, is characterized by insufficient production of thyroid hormones and is typically treated with oral levothyroxine (LT4). However, determining patient-specific LT4 dosage remains challenging due to significant inter-individual variability and the need for repeated clinical adjustments. In this study, a stability-aware, data-driven dosing framework is developed for personalized LT4 therapy. The proposed approach combines a Long Short-Term Memory (LSTM) neural network for predicting free thyroxine (FT4) concentrations with a model predictive control (MPC) strategy for dose optimization. To ensure safe and reliable operation, Input-to-State Stability (ISS) conditions are incorporated within the control framework, ensuring boundedness of the system states and stability of the closed-loop system. The method is evaluated using simulated patients generated with Thyrosim, a validated mathematical simulator of thyroid hormone regulation, under varying biological parameters. Simulation results demonstrate that the proposed framework regulates FT4 concentrations toward patient-specific euthyroid levels during the initial phase of treatment and maintains stable hormone levels throughout the simulation period. Furthermore, consistent performance across a heterogeneous population of simulated patients highlights the robustness of the approach. These findings suggest that the proposed ISS-LSTM-MPC framework provides a promising foundation for automated, personalized LT4 dosing and represents a step toward clinically applicable decision-support systems for thyroid hormone therapy.

## 1 Introduction

Hypothyroidism is a common endocrine disorder characterized by insufficient production of thyroid hormones, primarily thyroxine (T4) and triiodothyronine (T3). A gender-based association highlighted in indicates that hypothyroidism is more prevalent in females than in males, see e.g., Meng and et al (1). The condition affects approximately 10% of the general population, with nearly half of the affected individuals remaining undiagnosed. If left untreated, hypothyroidism may lead to fatigue, weight gain, cardiovascular complications, and metabolic dysfunction. In iodine-sufficient regions, Hashimoto’s thyroiditis is the leading cause of hypothyroidism, highlighted in Chiovato et al. (2).

Treatment of hypothyroidism is primarily based on oral administration of levothyroxine (LT4), which aims to restore thyroid hormone concentrations to a euthyroid state. However, determining an appropriate patient-specific LT4 dosage remains challenging due to significant inter-individual variability and the need for repeated dose adjustments during treatment. To understand the challenges associated with individualized LT4 dosing, it is important to consider the physiological regulation of thyroid hormones in the human body.

**Figure 1:**
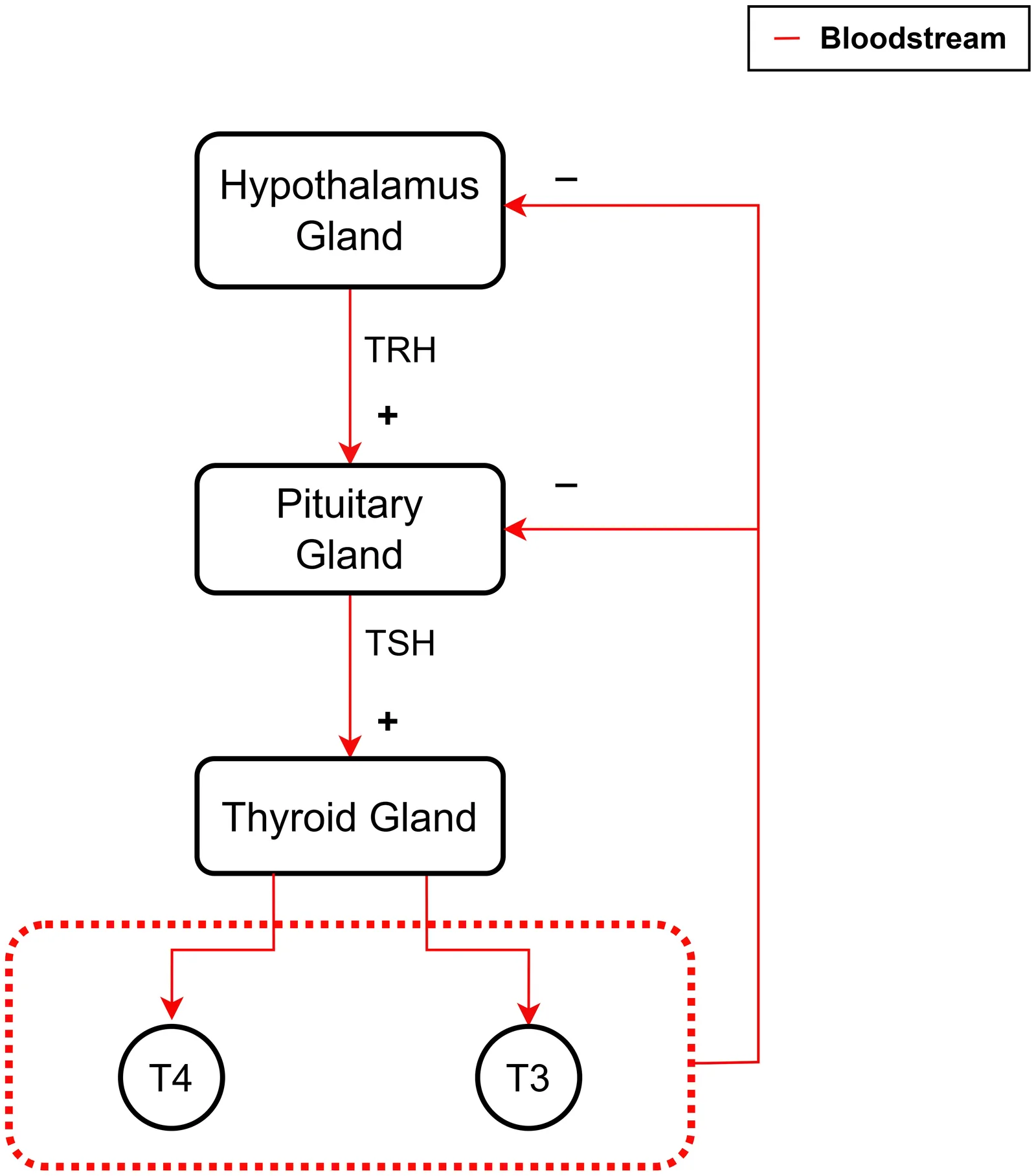
Block diagram of the hypothalamic–pituitary–thyroid (HPT) axis, illustrating thyroid hormone regulation in the human body.

The blood serum concentration of thyroid hormones is regulated in the human body by the hypothalamic–pituitary–thyroid (HPT) axis, see **Figure 1**. The concentrations of T4 and T3 act as negative feedback signals to the hypothalamus and pituitary gland (see e.g., Feldt-Rasmussen et al. (3)). In response, the pituitary and hypothalamus secrete thyroid-stimulating hormone (TSH) and thyrotropin-releasing hormone (TRH), respectively. As the levels of T4 and T3 increase, the biological feedback loop (HPT-axis) reduces TRH and TSH secretion, thereby maintaining hormonal balance.

### 1.1 Medical problem

The thyroid gland primarily consists of follicular cells, which are responsible for the production of T4 and T3 hormones. With the help of TSH, these follicular cells begin secreting thyroid hormones. In the case of Hashimoto’s thyroiditis, the immune system exerts autoimmune effects on the thyroid gland, leading to the production of thyroid peroxidase antibodies (TPOAb) and thyroglobulin antibodies (TgAb). These antibodies inhibit T4 and T3 hormone production within the follicular cells, see Fröhlich and Wahl (4). As a result, thyroid hormone concentrations fall below the levels required by the human body (i.e., T4: 5–12 *µ*g/dL and T3: 0.8–1.9 ng/mL, described in Eisenberg et al. (5)). The hypothalamus and pituitary gland respond by increasing TRH and TSH secretion via the HPT axis, leading to elevated TSH levels (0.5–5.0 mU/L, see e.g., Eisenberg et al. (5)). Patients with Hashimoto’s thyroiditis are therefore typically diagnosed with elevated TSH levels, reduced thyroid hormone concentrations, and increased levels of TPOAb and TgAb.

Thyroid hormones play a crucial role in regulating growth and energy metabolism. Consequently, disturbances in thyroid hormone concentrations can lead to various adverse health effects, including increased cardiovascular risk and weight gain. In severe cases, prolonged untreated hypothyroidism may result in myxedema coma.

In standard clinical practice, patients with hypothyroidism due to Hashimoto’s thyroiditis receive oral levothyroxine (LT4) therapy, typically administered once daily. The appropriate LT4 dosage is primarily adjusted based on serum thyroid-stimulating hormone (TSH) and free thyroxine (FT4) concentrations, together with clinical factors such as body weight, age, pregnancy status, and patient symptoms. Measurement of free triiodothyronine (FT3) is generally reserved for selected clinical situations and is not routinely used for LT4 dose adjustment. As a result, regular clinical visits are required for dose adjustment. The frequency of these visits depends on the disease stage, with more frequent (e.g., monthly) monitoring during the initial phase and less frequent follow-ups in later stages. However, maintaining stable thyroid hormone levels remains clinically challenging due to inter-individual variability in hormone absorption, metabolism, and disease progression. Consequently, a systematic and feedback-driven LT4 dosing strategy could improve long-term hormonal regulation compared to current trial-and-error approaches. Despite the widespread use of LT4 therapy, achieving adequate thyroid hormone replacement remains clinically challenging. A substantial proportion of patients receiving LT4 treatment are either overtreated or undertreated, which may contribute to adverse metabolic and cardiovascular outcomes, described in Efthymiadis et al. (6). These limitations further emphasize the need for personalized and feedback-driven dosing strategies capable of adapting to patient-specific physiological variability. In clinical practice, LT4 dosing strategies differ between overt and subclinical hypothyroidism. Patients with overt hypothyroidism are commonly treated using weight-based LT4 dosing approaches, whereas lower initial dosages are often prescribed in subclinical hypothyroidism depending on TSH elevation and clinical presentation, see Pilz et al. (7). Such conventional dosing approaches may serve as an important clinical reference for future comparisons with personalized computational dosing frameworks.

From a clinical perspective, an effective LT4 dosing strategy should maintain thyroid hormone concentrations within a patient-specific euthyroid range while remaining robust to inter-individual physiological variability. Avoiding excessive hormone fluctuations and prolonged hormonal imbalance is particularly important, as both may lead to adverse metabolic and cardiovascular effects. These clinical requirements motivate the development of a stability-aware, feedback-driven computational dosing framework, in which thyroid hormone measurements are used to adjust LT4 therapy, while mathematical stability conditions ensure reliable and bounded system behavior despite physiological variability.

This study aims to develop an automated and patient-specific LT4 dosing strategy that meets clinical dosing requirements (see Section 2.4). To achieve this, a Long Short-Term Memory (LSTM) neural network is utilized to predict FT4 concentrations based on Thyrosim simulation data. A stability-aware model predictive control (MPC) framework is then employed to determine appropriate LT4 dosages. Both the LSTM model and the MPC-based dosing strategy are validated using Thyrosim simulations (see Section 2.2). Such an approach has the potential to reduce the dependency on frequent in-person clinical visits and may serve as a decision-support tool to assist physicians in determining appropriate LT4 dosages.

Recently, machine learning has also been investigated for personalized LT4 dosage estimation using retrospective clinical data. For example, Ngan et al. (8) developed a machine learning-based predictive model for individualized LT4 dosage estimation from electronic medical records of hypothyroid patients. While such approaches provide personalized dosage recommendations, they do not explicitly consider the temporal dynamics of thyroid hormone regulation or closed-loop treatment optimization.

### 1.2 Prior approaches in stability-aware neural network MPC

Due to its optimization-based feedback structure, MPC has been widely used for controlling constrained systems. In recent years, deep learning models, particularly LSTM networks, have gained attention due to their ability to model complex nonlinear dynamics from data. While neural networks offer flexibility and strong predictive capabilities, their integration into closed-loop control systems raises important concerns regarding stability and robustness.

In a physiological context, closed-loop stability refers to the ability of the system to restore hormonal equilibrium after disturbances, ensuring that TSH and FT4 concentrations return to their normal ranges under the recommended LT4 dosing. Robustness, in turn, ensures that this equilibrium is maintained despite inter-individual variations in biological parameters, such as hormone kinetics and drug absorption rates.

The integration of LSTM networks into MPC frameworks has been investigated in Terzi et al. (9), where Input-to-State Stability (ISS) properties were used to analyze stability. However, these stability properties were enforced during offline training and not directly incorporated into the MPC optimization problem. Similarly, Jung et al. (10) demonstrated improved prediction and control performance using LSTM-based MPC, but without formal stability guarantees.

In Stiti et al. (11), Lyapunov-based constraints were incorporated into an MPC framework to ensure closed-loop stability, although the Lyapunov function was derived from known system dynamics rather than the neural network itself. In contrast, the present study derives stability conditions directly from the internal dynamics of the LSTM network, enabling stability-aware control of a data-driven model.

Recent advances in biomedical control further highlight the relevance of combining predictive models with control strategies. Applications include glucose regulation in diabetes Sun et al. (12), anesthesia control Pawłowski et al. (13, 14), and predictive monitoring systems Iacono et al. (15). These developments motivate the use of data-driven yet stability-aware approaches for personalized hormone therapy.

In this study, we propose an ISS-constrained LSTM-MPC framework in which Lyapunov-based stability conditions are incorporated directly into the MPC optimization problem. These conditions are applied both during network training and control, ensuring stable closed-loop behavior while leveraging the predictive capabilities of LSTM networks. To the best of the authors’ knowledge, this is the first study to integrate ISS-based stability guarantees directly within an LSTM-MPC framework for thyroid hormone regulation.

### 1.3 Existing control-oriented levothyroxine dosing strategies

In Sharma et al. (16), an MPC-based LT4 dosing strategy was developed using a reduced-order mathematical model of thyroid hormone regulation Sharma et al. (17). The approach incorporates individualized euthyroid reference levels, estimated from FT4 and TSH measurements using the set-point theory, and optimizes LT4 dosing with respect to this personalized target while ensuring operation within predefined constraints. Similar to this model-based approach, the proposed ISS-LSTM-MPC framework individualizes LT4 dosing by regulating each patient’s FT4 concentration toward the personalized euthyroid reference obtained using the set-point theory.

In Wolff et al. (18), optimal LT4 and LT3 combination therapy was investigated using a detailed physiological model. Such mechanistic approaches capture complex thyroid hormone dynamics and provide a physiologically interpretable framework for therapy optimization. However, their computational complexity may limit their direct integration into computationally efficient optimization-based controllers. Therefore, in this work, an LSTM surrogate model trained using Thyrosim simulations is employed within an MPC framework to provide computationally efficient FT4 prediction while maintaining physiologically relevant dynamics.

Recently, personalized mechanistic models have also been proposed for individualized thyroid hormone replacement therapy. Cruz-Loya et al. (19) introduced the personalized p-THYROSIM model by extending the original Thyrosim framework to account for patient-specific anthropometric characteristics, enabling individualized optimization of LT4 and LT4+LT3 replacement therapy. Subsequently, DiStefano et al. (20) further refined p-THYROSIM by improving the personalization strategy and providing an iOS implementation to facilitate its use as a clinical decision-support tool. These studies demonstrate the potential of personalized mechanistic modeling for thyroid hormone replacement therapy. Building upon these developments, the proposed framework utilizes personalized mechanistic modeling for the automatic computation of personalized daily LT4 dosing recommendations. Simulation data generated by the mechanistic Thyrosim model are used to train an ISS-aware LSTM-based FT4 prediction model, which is subsequently integrated into an ISS-constrained MPC framework for closed-loop personalized LT4 dosing.

Despite these advances, existing LT4 dosing strategies often rely on predefined physiological models and lack stability-aware, data-driven closed-loop solutions. In particular, ensuring safe and reliable performance across a wide range of patients remains a key challenge.

In this work, we propose a stability-aware LSTM-MPC framework for personalized LT4 dosing in patients with Hashimoto’s thyroiditis. The framework ensures reliable closed-loop behavior through the incorporation of stability conditions, integrates clinically relevant constraints such as dosage limits and euthyroid bounds, and demonstrates robustness across multiple simulated patients using Thyrosim, see Section 2.2.

### 1.4 Overall workflow of the proposed framework

**Figure 2:**
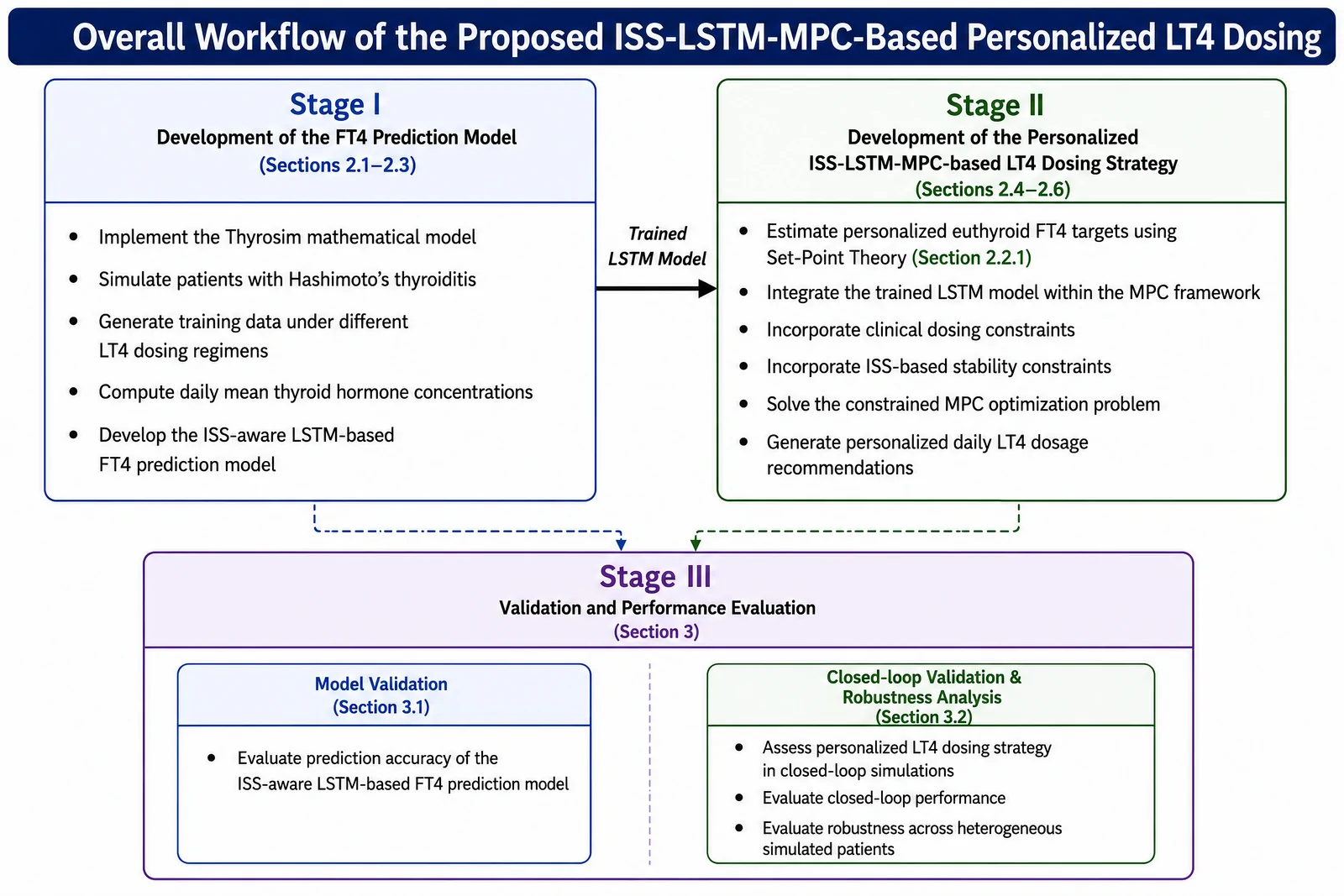
Overall workflow of the proposed ISS-LSTM-MPC-based personalized LT4 dosing framework. Stage I develops the ISS-aware LSTM-based FT4 prediction model using simulation data generated by the Thyrosim mathematical model. Stage II integrates the trained LSTM model into an ISS-constrained MPC framework to generate personalized LT4 dosing recommendations based on patient-specific euthyroid reference concentrations. Stage III evaluates the proposed framework through model validation, closed-loop controller validation, and robustness analysis.

**Figure 2** provides an overview of the proposed ISS-LSTM-MPC-based personalized LT4 dosing framework. The methodology consists of three main stages. First, simulation data generated using the Thyrosim mathematical model are used to develop the ISS-aware LSTM-based FT4 prediction model. Second, the trained LSTM model is integrated into an ISS-constrained MPC framework together with patient-specific euthyroid reference concentrations estimated using the set-point theory to generate personalized LT4 dosing recommendations. Finally, the proposed framework is evaluated through model validation, closed-loop validation, and robustness analysis. The details of each stage are presented in the following sections.

## 2 Methods

For clarity and consistency, the notation used throughout this work is summarized in **Supplementary Section S1**.

This section describes the methodology used to develop the proposed ISS-LSTM-MPC-based LT4 dosing strategy for patients suffering from Hashimoto’s thyroiditis. We first provide an overview of the proposed framework, followed by descriptions of the simulation model, the LSTM-based FT4 prediction model, and the MPC-based dosing strategy. Thyrosim is employed as a simulated patient model, and the overall framework is evaluated through numerical simulations to assess performance and robustness.

### 2.1 Overview of the proposed ISS-LSTM-MPC framework

This section provides an overview of the proposed personalized LT4 dosing framework. The objective of this study is to design a patient-specific LT4 dosing strategy for individuals with Hashimoto’s thyroiditis. To this end, an LSTM-based FT4 prediction model is developed to capture the underlying thyroid hormone dynamics. Based on this prediction model, an MPC framework is employed to determine appropriate LT4 dosages.

To ensure safe and reliable operation, stability considerations based on ISS are incorporated for both the LSTM model and the closed-loop system. The resulting framework functions as an LT4 dosing recommendation system. Thyrosim is used as a simulated patient environment to validate both the prediction model and the dosing strategy.

### 2.2 Mathematical simulation of a patient suffering from Hashimoto’s thyroiditis

Thyrosim^1^ is a web application developed for researchers and clinicians interested in the analysis of thyroid hormone regulation. It represents a well-validated mathematical model of human thyroid hormone dynamics. In the proposed framework, the Thyrosim model presented in Eisenberg et al. (21) Eisenberg and Distefano (22) Han et al. (23), and Eisenberg et al. (5) is used to simulate patients. Thyrosim has been shown to reproduce a wide range of clinical data and captures dynamic hormonal responses to different oral dosages of LT4 and LT3.

The Thyrosim mathematical model consists of 13 coupled differential equations and approximately 56 physiological model parameters describing thyroid hormone regulation. The model was implemented in MATLAB/Simulink to serve as the simulated patient throughout this study. Although this detailed physiological model accurately represents thyroid hormone regulation, its direct integration into an optimization-based LT4 dosing strategy is computationally demanding. Therefore, this work adopts a hybrid modeling approach, in which simulation data generated by Thyrosim are used to train an LSTM-based FT4 prediction model. The trained LSTM-based FT4 prediction model is subsequently employed within the MPC framework for computationally efficient FT4 prediction, while the full Thyrosim model is retained as the mechanistic reference model for validating both the prediction model and the dosing algorithm.

To simulate patients with clinical hypothyroidism, the T4 and T3 secretion-related parameters within the Thyrosim model are reduced relative to nominal healthy values. For the nominal simulated patient, the secretion rates are set to 17% of the thyroid gland’s total secretion capacity, indicating dependence on exogenous hormone administration. During robustness evaluation and statistical analysis, these and other biological parameters are varied within their physiological ranges to generate heterogeneous simulated patients with differing thyroid hormone dynamics. Correspondingly, the absorption rate of orally administered LT4 is set at 88%, consistent with the original Thyrosim model Eisenberg et al. (5).

#### 2.2.1 Estimation of personalized healthy thyroid hormone concentration

In Goede (24) and Leow and Goede (25), a so-called set-point theory is introduced to estimate patient-specific healthy thyroid hormone concentrations, such as FT4 and TSH. This method can be applied during the early phase of treatment to determine individualized euthyroid targets. These targets can subsequently be achieved through appropriate LT4 dosing. The estimation of patient-specific healthy thyroid hormone concentrations from measured FT4 and TSH values may reduce the frequency of clinical consultations compared with current practice, where repeated hormone measurements (e.g., every 6–8 weeks initially and twice yearly thereafter) are required.

To determine the patient-specific set points [FT4]_sp_ and [TSH]_sp_, measurements of FT4 and TSH obtained during treatment are used to characterize the patient’s individual hypothalamus–pituitary (HP) relationship. At least two distinct measurements, i.e., [*FT*4]_1_, [*FT*4]_2_, [*TSH*]_1_, and [*TSH*]_2_, are required. Using the parameters *S* and *φ*, the patient’s HP characteristics are described by Equations 1a–1d:

(1a) $$\varphi =\left(\frac{1}{[FT4{]}_{1}-[FT4{]}_{2}}\right)\ln\frac{{\left[TSH\right]}_{2}}{{\left[TSH\right]}_{1}},$$

(1b) $$S=[TSH{]}_{1}\exp\;(\varphi [FT4{]}_{1}),$$

the set point values are given by [*FT*4]*_sp_* and [*TSH*]*_sp_*,

(1c) $${\left[FT4\right]}_{sp}=\frac{1}{\varphi }\ln\;(\varphi S\sqrt{2}),$$

(1d) $${\left[TSH\right]}_{sp}=\frac{1}{\varphi \sqrt{2}}.$$

In this study, the set-point theory (1) is applied to estimate patient-specific healthy thyroid hormone concentrations from individual FT4 and TSH measurements. These estimated euthyroid concentrations serve as the personalized reference for the proposed ISS-LSTM-MPC-based LT4 dosing strategy. The controller is then implemented on simulated patients (emulated using Thyrosim) to regulate FT4 concentrations toward these individualized targets.

### 2.3 LSTM-based FT4 prediction model

An LSTM neural network is a subtype of recurrent neural networks, capable of capturing long-term temporal dependencies Hochreiter and Schmidhuber (26). This property is particularly useful for modeling the dynamic relationship between LT4 dosing and FT4 concentrations. Furthermore, Bruni et al. (27) highlighted that LSTM networks are robust to input noise and disturbances, making them suitable for biological systems where patient-specific parameters may vary.

Simulation data generated from Thyrosim are used to train the LSTM neural network. It is assumed that the underlying system dynamics are not explicitly known, and only measurements of relevant thyroid parameters are available. During training, the physiological parameters of the Thyrosim mathematical model were maintained at their nominal values. To assess robustness, the patient-specific parameters were subsequently varied according to their reported coefficients of variation (CVs), thereby generating a heterogeneous population of simulated patients with corresponding changes in FT4 dynamics. Specifically, each patient-specific parameter *P* was perturbed relative to its nominal value according to its reported CV, i.e., $P=P_{nom}\left(1\pm \frac{CV}{100}\right)$, where *P*_nom_ represents the nominal value of the corresponding patient-specific parameter. The nominal parameter values and their associated CVs are provided in **Supplementary Table 1** in **Section S5 of the Supplementary Material**, reproduced from Han et al. (23). Despite these parameter variations, the LSTM-based FT4 prediction model accurately predicts FT4 concentrations, closely matching the responses of the simulated patients. This predictive capability is essential for designing the MPC-based dosing strategy and ensuring reliable LT4 recommendations across different patient scenarios.

#### 2.3.1 ISS-LSTM neural network-based FT4 prediction

In Bonassi et al. (28) analyzed LSTM neural networks from a dynamical systems perspective and derived sufficient conditions for ensuring ISS. In this context, the LSTM model can be represented as a discrete-time nonlinear dynamical system given by Equations 2a, b:

(2a) $$\chi^{+}=f_{\text{LSTM}}\left(\chi ,u,b_{c}\right),$$

(2b) $$y=g_{\text{LSTM}}\left(\chi \right),$$

where *χ* represents the internal LSTM states (hidden and cell states), *u* denotes the input, *y* is the predicted FT4 concentration and *b_c_* denotes the bias vector associated with the candidate gate of the LSTM network. When viewed in this form, ISS ensures that the internal states remain bounded under bounded inputs, which is essential for stable and reliable predictions.

These ISS-based considerations are incorporated in this study to enhance the stability of the LSTM-based FT4 prediction model. From a physiological perspective, thyroid hormone concentrations in the human body remain within bounded ranges under normal regulatory mechanisms. Therefore, incorporating ISS-based properties into the LSTM model is consistent with these biological constraints and supports the development of reliable prediction models for clinical applications.

A more detailed mathematical formulation of the LSTM network architecture is provided in **Section S2 of the Supplementary Material**.

#### 2.3.2 Input-to-state stability of LSTM networks

In this study, the ISS conditions derived by Bonassi et al. (28) are employed to ensure stability of the LSTM-based FT4 prediction model. Specifically, if the discrete-time, nonlinear dynamical system (2) admits a time-invariant ISS-Lyapunov function *V* such that there exist functions $\psi_{1},\psi_{2},\psi \in K_{\infty }$ and $\sigma_{u},\sigma_{b},\in K$ satisfying Equations 3a–3b:

(3a) $$\psi_{1}({\left|\chi \right|}_{2})\leq V\left(\chi \right)\leq \psi_{2}({\left|\chi \right|}_{2}),$$

(3b) $$V\left(f_{\text{LSTM}}\left(\chi ,u,b_{c}\right)\right)-V\left(\chi \right)\leq -\psi ({\left|\chi \right|}_{2})+\sigma_{u}({\left|u\right|}_{2})+\sigma_{b}({\left|b_{c}\right|}_{2}),$$

then the system is input-to-state stable. These conditions ensure that the internal states of the LSTM network remain bounded under bounded inputs, thereby supporting stable and reliable predictions.

The detailed derivation and mathematical expressions of the ISS-based stability conditions are provided in **Section S3 of the Supplementary Material**. In the present work, these formulations are used to ensure bounded and reliable behavior of the data-driven prediction model.

#### 2.3.3 Generating training data for LSTM neural network

In this study, Thyrosim was employed to simulate patients suffering from Hashimoto’s thyroiditis. The Thyrosim model was simulated over 160 days under different daily LT4 dosing regimens. For each simulated day, Thyrosim generated the corresponding TSH, T3, T4, and FT4 responses. These simulated hormone measurements were averaged over each day to generate the training dataset for the LSTM neural network. Consequently, the LSTM learns the relationship between daily LT4 administration and the resulting FT4 concentration from simulation data generated by the physiologically based Thyrosim model. The resulting discrete-time dataset has a sampling interval of 24 hours.

The LSTM neural network consists of three input signals (*n_u_* = 3), namely the daily mean plasma concentration of T4, the daily mean amount of LT4 available for absorption within the gastrointestinal compartment of the Thyrosim model, and the daily oral LT4 dosage. The inclusion of the gastrointestinal LT4 compartment enables the network to capture delayed absorption dynamics associated with oral hormone administration. The model produces a single output corresponding to the daily mean FT4 concentration.

It is assumed that the patient is suffering from clinical hypothyroidism. Therefore, after the initial treatment phase, the total FT4 concentration is primarily driven by exogenous LT4 administration. Consequently, the effect of TSH on FT4 dynamics is assumed to be minimal and is not explicitly included in the training of the LSTM model.

#### 2.3.4 LSTM network training

To minimize the total loss, the Adam optimizer, described in Kingma and Ba (29) is employed with an adaptive learning rate. The total loss function *L* is defined by Equation 4:

(4) $$L=\lambda_{1}L_{\text{P}}+\lambda_{2}L_{\text{I}}+\lambda_{3}L_{\text{R}},$$

where, *L*_P_ is defined by the prediction error, *L*_I_ is defined by the penalty for violating ISS conditions, and *L*_R_ is given by a regularization term on model parameters. The hyperparameters *λ*_1_, *λ*_2_, *λ*_3_ are the positive weighting coefficients. The network training balances prediction accuracy and stability by minimizing the total loss *L*, with the Adam optimizer ensuring efficient convergence.

##### 2.3.4.1 Training implementation

ISS-based stability considerations are incorporated through an additional loss term that penalizes violations of stability conditions (see **Section S3 in the Supplementary Material**). The term *L*_R_ is employed to penalize certain model parameters to prevent overfitting. The minimization of *L*_I_ and *L*_R_ encourages the trained network to satisfy the desired stability properties. A single-layer LSTM network with 450 hidden units (*n_x_* = 450), followed by a flattened output layer, was implemented to predict FT4 levels from a time-series sequence of 160 daily measurements, each containing 3 input features *u* (the daily mean pool plasma concentration of T4, the daily mean of LT4 available for absorption within the gastrointestinal compartment, and the daily oral intake of LT4 medication).

The network was trained with an initial learning rate of 0.005 and default hyperparameters (*β*_1_ = 0.9, *β*_2_ = 0.999, *ϵ* = 10^−8^). The loss function was weighted using *λ*_1_ = 1, *λ*_2_ = 10, and *λ*_3_ = 10, with the weights selected to balance stability enforcement and prediction accuracy during training. Training ran for 1000 epochs with a batch size of 1 in MATLAB. The optimization successfully minimized the total loss to *L<* 0.01, confirming both stability enforcement and accurate predictive performance.

### 2.4 Dosing strategy requirements

The design of the LT4 dosing strategy must account for several clinically relevant considerations. First, patient-specific biological parameters may vary significantly between individuals, requiring a robust dosing approach. Second, oral administration of T4 (i.e., LT4 dosage) can lead to sudden increases in blood serum FT4 concentration; therefore, a rate limiter is introduced to restrict the maximum admissible change in the recommended dosage (i.e., 25 *µ*g). Third, any dosage exceeding 200 *µ*g/day typically requires physician supervision, and thus the recommended dose is constrained by this clinically accepted upper limit. Furthermore, since patients are instructed to take a single LT4 dose per day, a discrete-time dosing scheme with a 24-hour sampling interval is adopted. Finally, the patient’s FT4 concentration must remain within the clinically accepted euthyroid range throughout treatment, which is enforced through output constraints.

Similar clinical input and output constraints are commonly employed in mechanistic model-based LT4 dosing strategies (e.g., those employing Thyrosim) to ensure safe hormone regulation and clinically acceptable dosing. The presence of bounded FT4 levels, limits on the maximum LT4 dosage, and restrictions on dosage variation highlights a key advantage of using an MPC-based dosing strategy. MPC enables the systematic incorporation of these constraints to ensure that dosing recommendations remain within predefined clinical safety limits.

This capability is particularly important in biomedical applications, where precise dosing and adherence to clinical safety requirements are essential. From a clinical perspective, stability corresponds to maintaining a patient-specific euthyroid state, while robustness reflects the reliability of the therapy across diverse patient profiles. These properties are critical for avoiding hormone overshoot and prolonged imbalance, both of which may lead to adverse physiological effects.

### 2.5 ISS-LSTM-MPC-based LT4 dosing strategy

The prediction horizon Np is selected for two weeks (i.e., fourteen days) and the control horizon Nc is selected for a week (i.e., seven days). The output *y* is predicted over the prediction horizon Np, the predicted output signals $y_{k+1},\;y_{k+2},\ldots ,y_{k+Np}$ can be written as predicted output vector defined in Equation 5a:

(5a) $$y^{T}=\left[y_{k+1}\;y_{k+2}\;\ldots \;y_{k+Np}\right].$$

Similarly, the recommended LT4 dosage *d* and the change in the recommended LT4 dosage Δ*d* can be represented as by the vectors defined in Equations 5b and 5c:

(5b) $$\;d^{T}=\left[d_{k}\;d_{k+1}\;\ldots \;d_{k+Nc-1}\right],$$

(5c) $$\text{Δ}d^{T}=\left[\text{Δ}d_{k}\;\text{Δ}d_{k+1}\;\ldots \;\text{Δ}d_{k+Nc-1}\right],$$

The reference signal *r* is constant throughout the prediction horizon Np and can be written as the vector defined in Equation 5d:

(5d) $$r^{T}=\left[r_{k+1}\;r_{k+2}\;\ldots \;r_{k+Np}\right].$$

In order to ensure that a feasible solution exist within an acceptable operating range with respect to necessary performance limitations, several constraints are proposed. The proposed ISS-LSTM-MPC-based LT4 dosing strategy incorporates input, output and additionally, Lyapunov function-based constraints, see Section 2.3.2. To find the optimal control sequence, a quadratic optimization problem is solved. The recommended LT4 dosage *d* is optimized over the control horizon Nc by solving the quadratic optimization problem given by Equations 6a–6g:

(6a) $$\min_{d}\left(e^{T}Qe+\text{Δ}d^{T}R\text{Δ}d\right)$$

subject to,

(6b) $$d_{min}\leq d\leq d_{max},$$

(6c) $$\text{Δ}d_{min}\leq \text{Δ}d\leq \text{Δ}d_{max},$$

(6d) $$y_{min}\leq y\leq y_{max},$$

(6e) $$\psi_{1}({\left|\chi \right|}_{2})\leq V\left(\chi \right)\leq \psi_{2}({\left|\chi \right|}_{2}),$$

(6f) $$V\left(f_{\text{LSTM}}\left(\chi ,u,b_{c}\right)\right)-V\left(\chi \right)\leq -\psi \left({\left|\chi \right|}_{2}\right)+\sigma_{u}\left({\left|u\right|}_{2}\right)+\sigma_{b}\left({\left|b_{c}\right|}_{2}\right).$$

The vector *e* represents the error between the predicted output *y* and the reference signal *r* i.e.,

(6g) e=y−r.

The objective of this study is to maintain patient-specific euthyroid level (e.g., healthy concentration of thyroid hormones) in the patient’s body, while ensuring the closed-loop stability of the developed dosing algorithm. To achieve this, ISS conditions derived by Bonassi et al. (28), explained in **Supplementary Material**, are incorporated as constraints for solving a quadratic optimization problem. The controller leverages the trained LSTM model to predict FT4 levels and optimizes daily LT4 dosages by minimizing the quadratic cost function, s.t., stability constraints from Equations 3, where a feasible solution ensures the system operates within a predefined stability region satisfying ISS. Additionally, the change in LT4 dosage Δ*d* is limited by the minimum change Δ*d*_min_ and the maximum change Δ*d*_max_, set to ± 25 µg. This constraint reflects standard clinical practice, where LT4 dose adjustments are typically performed in increments of 12.5–25 µg, thereby avoiding unnecessarily large dosage modifications between treatment assessments. Furthermore, the maximum dosage *d_max_* is capped at 200 µg and the minimum dosage *d_min_* is set to 0 µg, as the dosage exceeding 200 µg require consultation with a physician. In the study by Park et al. (30), the range of euthyroid state specifies that the FT4 concentration *y* is constrained to *y_min_* = 9.2 ng/L to *y_max_* = 16 ng/L, to maintain a healthy thyroid state throughout the simulated operations.

The developed ISS-LSTM-MPC-based LT4 dosing strategy ensures that patient FT4 levels remain within predefined biological limits while operating within a stability region satisfying ISS. Utilizing an LSTM model to predict FT4 levels, the strategy optimizes daily LT4 dosages, maintaining euthyroid levels and ensuring closed-loop stability.

A detailed proof of closed-loop stability for the proposed framework is provided in **Section S4 of the Supplementary Material**.

### 2.6 Control algorithm

The proposed ISS-LSTM-MPC-based LT4 dosing strategy is implemented as follows:

Step 1: Initialization.

- Define the prediction horizon *N_p_*, control horizon *N_c_*, and initial LT4 dosage *d*_0_.
- Set the patient-specific FT4 reference trajectory over the prediction horizon.

Step 2: Optimization.

- Predict FT4 concentrations using the trained LSTM model.
- Solve the constrained optimization problem to determine the optimal LT4 dosage sequence, incorporating clinical constraints and ISS-based stability conditions.

Step 3: Dose administration.

- Apply the first element of the optimized LT4 dosage sequence.
- Update the system state and repeat the procedure at the next time step.

The detailed implementation of ISS-based constraints and their numerical evaluation within the optimization procedure are provided in Section S3 and Section S4 of the Supplementary Material.

## 3 Results

In this section, the developed LSTM-based FT4 prediction model and ISS-LSTM-MPC-based LT4 dosing strategy were implemented with Thyrosim to evaluate their performance. Simulation-based results are presented to demonstrate the effectiveness of the proposed approach.

The results of this study were obtained using MATLAB/Simulink^®^, 9.13.0.2399474 (R2022b) on a standard computer (Intel(R) Core(TM) i7-8565U CPU @ 1.80GHz 1.99 GHz processor with 16.0 GB).

### 3.1 Model validation

**Figure 3:**
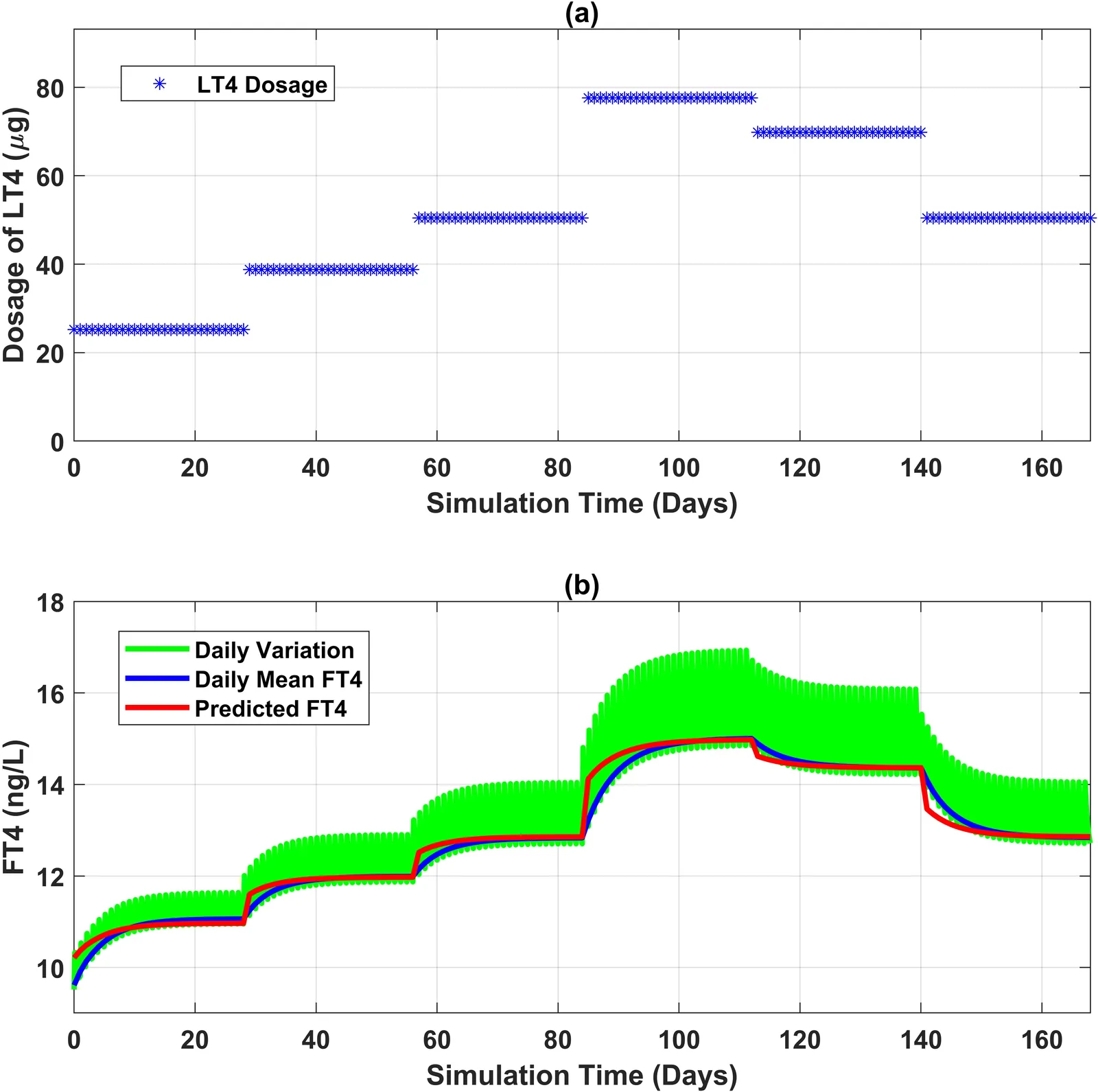
Validation of the proposed LSTM-based FT4 prediction model. **(a)** LT4 dosing regimen applied to both the Thyrosim model and the proposed LSTM model. **(b)** Comparison between the continuous FT4 response generated by the Thyrosim model (green), the corresponding daily mean FT4 concentration (blue), and the FT4 concentration predicted by the proposed LSTM model (red).

A comparison between the FT4 concentration generated by the LSTM-based FT4 prediction model and Thyrosim is presented to assess the quality of proposed LSTM-based FT4 prediction model (2). In the assessment, we provided the same amount of dosage to Thyrosim and the proposed LSTM-based FT4 prediction model. In **Figure 3a**, both mathematical models received multiple different oral dosages of LT4, varies from 25 *µ*g to 80 *µ*g. Thyrosim also captures daily hormonal variations of thyroid hormone parameters. Note that the proposed model (2) does not replicate these daily hormonal variations in FT4 concentration, see **Figure 3b**, as the LSTM neural network trained with daily mean of thyroid parameters, (see Section 2.3.3). From the results presented in **Figure 3b**, it is noticeable that the generated FT4 concentration by the proposed LSTM model (2) always lies within the daily hormonal variation of FT4 concentration generated by Thyrosim and remains close to the daily mean of FT4 concentration. As a result, the proposed framework represents a hybrid modeling approach, in which the LSTM-based FT4 prediction model is integrated into the ISS-LSTM-MPC framework for computationally efficient personalized LT4 dosing, while the mechanistic Thyrosim model is used as the physiological reference model for validation.

### 3.2 Validation of developed ISS-LSTM-MPC-based LT4 dosing strategy

Different simulated patients were generated by varying the patient-specific biological parameters of the Thyrosim mathematical model according to their reported CVs, as described in Section 2.3. All the simulated patients are treated with the developed ISS-LSTM-MPC based LT4 dosing strategy. The developed system should be compatible with the possible parametric uncertainties and should be able to maintain patient’s FT4 concentration within the reference range.

#### 3.2.1 Simulation results

The MPC cost function utilizes a weighting matrix $Q=I_{N_{P}}$ to penalize deviations in FT4 tracking over the prediction horizon, and a regularization term $R=5000\cdot I_{\mathrm{Nc}}$ to penalize changes in LT4 dosage over the control horizon.

**Figure 4:**
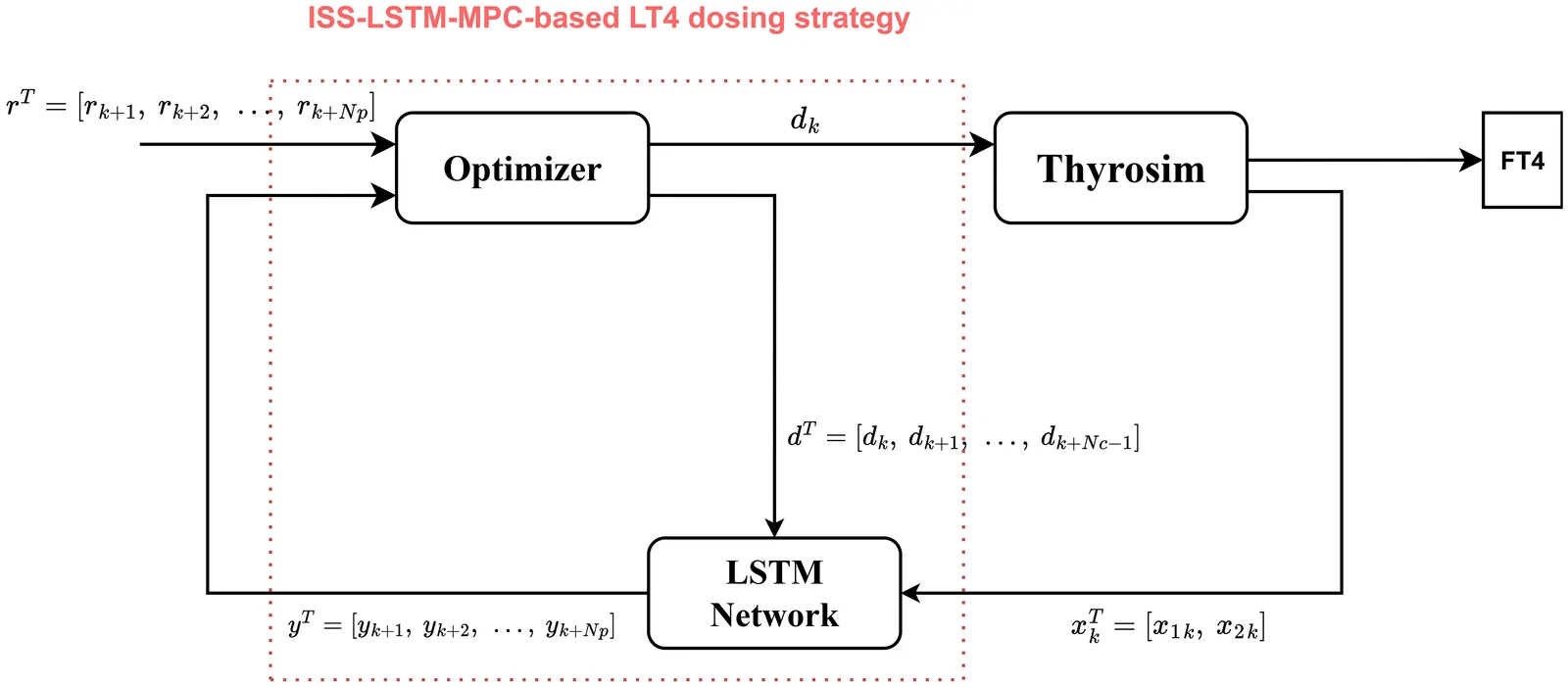
Structure of the implemented simulation setup for evaluating the ISS-LSTM-MPC-based LT4 dosing strategy.

The developed ISS-LSTM-MPC-based LT4 dosing strategy is applied to regulate the FT4 concentration of a patient simulated using Thyrosim (see **Figure 4**).

**Figure 5:**
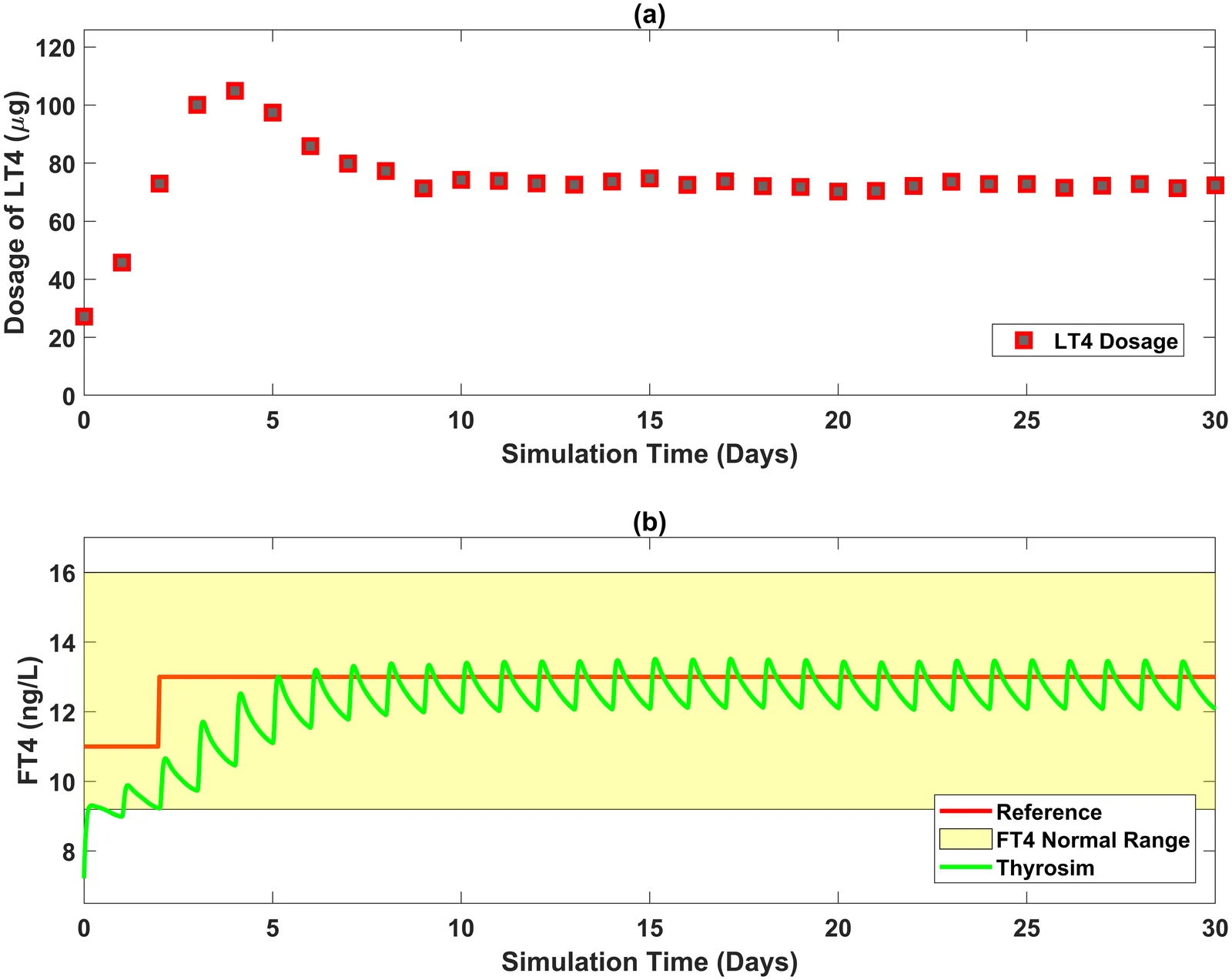
Simulation results of the proposed ISS-LSTM-MPC-based LT4 dosing strategy for a simulated patient over a 30-day period. **(a)** Recommended LT4 dosage profile with a maximum admissible dosage change of 25 μg. **(b)** Corresponding FT4 concentration, with the reference initially set to 11 ng/L and updated after three days to the patient-specific euthyroid level of 13.1 ng/L.

**Figure 5a** shows the LT4 dosage recommended by the proposed strategy, where a maximum admissible change of 25 *µ*g is enforced. The biological parameters of Thyrosim are varied to represent a simulated patient with altered thyroid dynamics. **Figure 5b** presents the corresponding FT4 concentration. The total simulation duration is 30 days. A patient-specific healthy FT4 concentration is estimated using the set-point theory described in Section 2.2.1. The initial reference FT4 concentration is set to 11 ng/L and updated after three days to the personalized euthyroid level (13.1 ng/L). The proposed ISS-LSTM-MPC-based dosing strategy adjusts the LT4 dosage such that the FT4 concentration converges to the desired range within approximately one week. Thereafter, the FT4 concentration remains within the patient-specific euthyroid range for the remainder of the simulation period.

**Figure 6:**
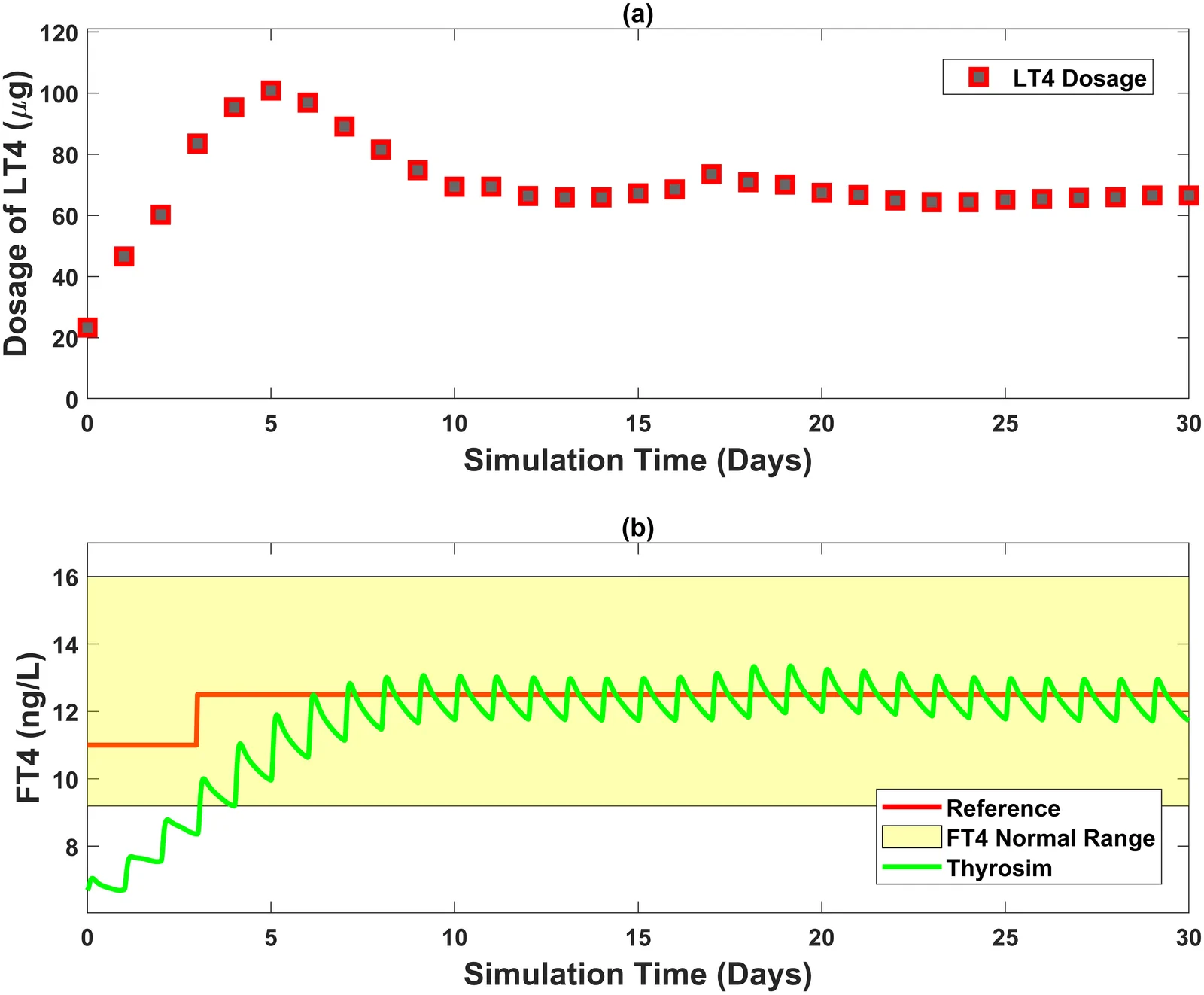
Simulation results of the proposed ISS-LSTM-MPC-based LT4 dosing strategy for a second simulated patient over a 30-day period. **(a)** Recommended LT4 dosage profile. **(b)** Corresponding FT4 concentration, with the reference initially set to 11 ng/L and updated after three days to the patient-specific euthyroid level of 12.6 ng/L.

To further evaluate robustness, a second simulated patient is generated by varying Thyrosim’s biological parameters. **Figure 6a** shows the corresponding LT4 dosage profile, while **Figure 6b** presents the FT4 concentration response. A patient-specific FT4 reference is estimated using the set-point theory. The initial reference (11 ng/L) is updated after three days to 12.6 ng/L. The proposed dosing strategy successfully regulates FT4 levels to the desired range within one week and maintains personalized euthyroid hormone levels throughout the 30-day simulation.

**Figure 7:**
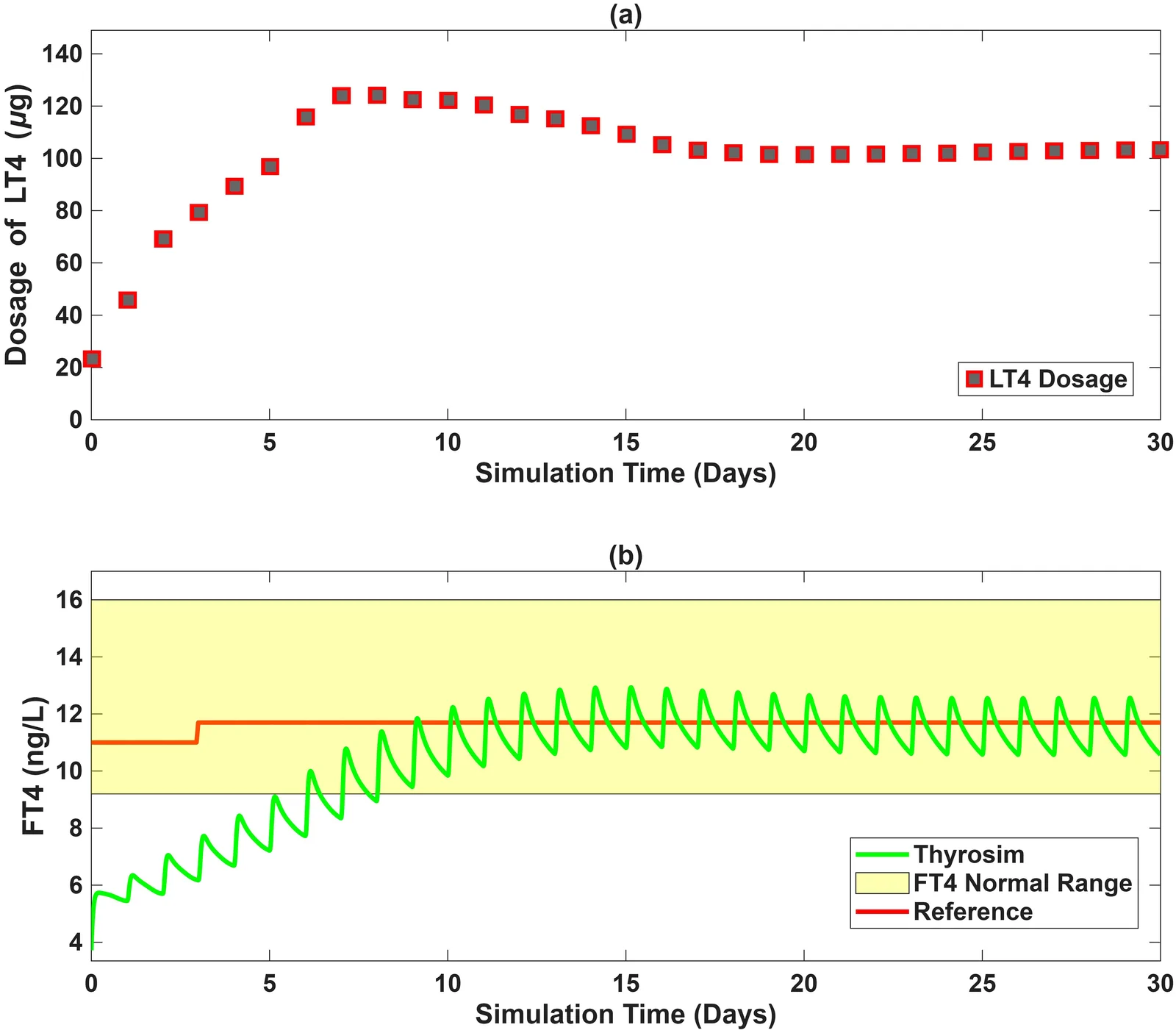
FT4 regulation for a third simulated patient with altered biological parameters, resulting in a lower initial FT4 concentration (4 ng/L) than the previously presented simulated patients, over a 30-day simulation period. **(a)** Recommended LT4 dosage profile. **(b)** Corresponding FT4 concentration, with the reference initially set to 11 ng/L and updated after three days to the patient-specific euthyroid level of 11.6 ng/L.

As an additional simulation study, a third simulated patient is generated by varying Thyrosim’s biological parameters. **Figure 7a** shows the corresponding LT4 dosage profile, while **Figure 7b** presents the FT4 concentration response. A patient-specific FT4 reference is estimated using the set-point theory. The initial reference (11 ng/L) is updated after three days to 11.6 ng/L. The simulated patient has an initial FT4 concentration of 4 ng/L. The proposed dosing strategy successfully regulates the FT4 concentration to the personalized euthyroid reference within two weeks and maintains the personalized euthyroid hormone levels throughout the remainder of the 30-day simulation period.

These individual simulations illustrate the effectiveness and robustness of the proposed dosing strategy under varying patient conditions.

##### 3.2.1.1 Statistical analysis

To further evaluate the robustness of the proposed approach, a population-level analysis is performed. A heterogeneous population of 50 simulated patients is generated by varying the patient-specific physiological parameters of the Thyrosim mathematical model according to their reported CVs, following the procedure described in Section 2.3.

**Figure 8:**
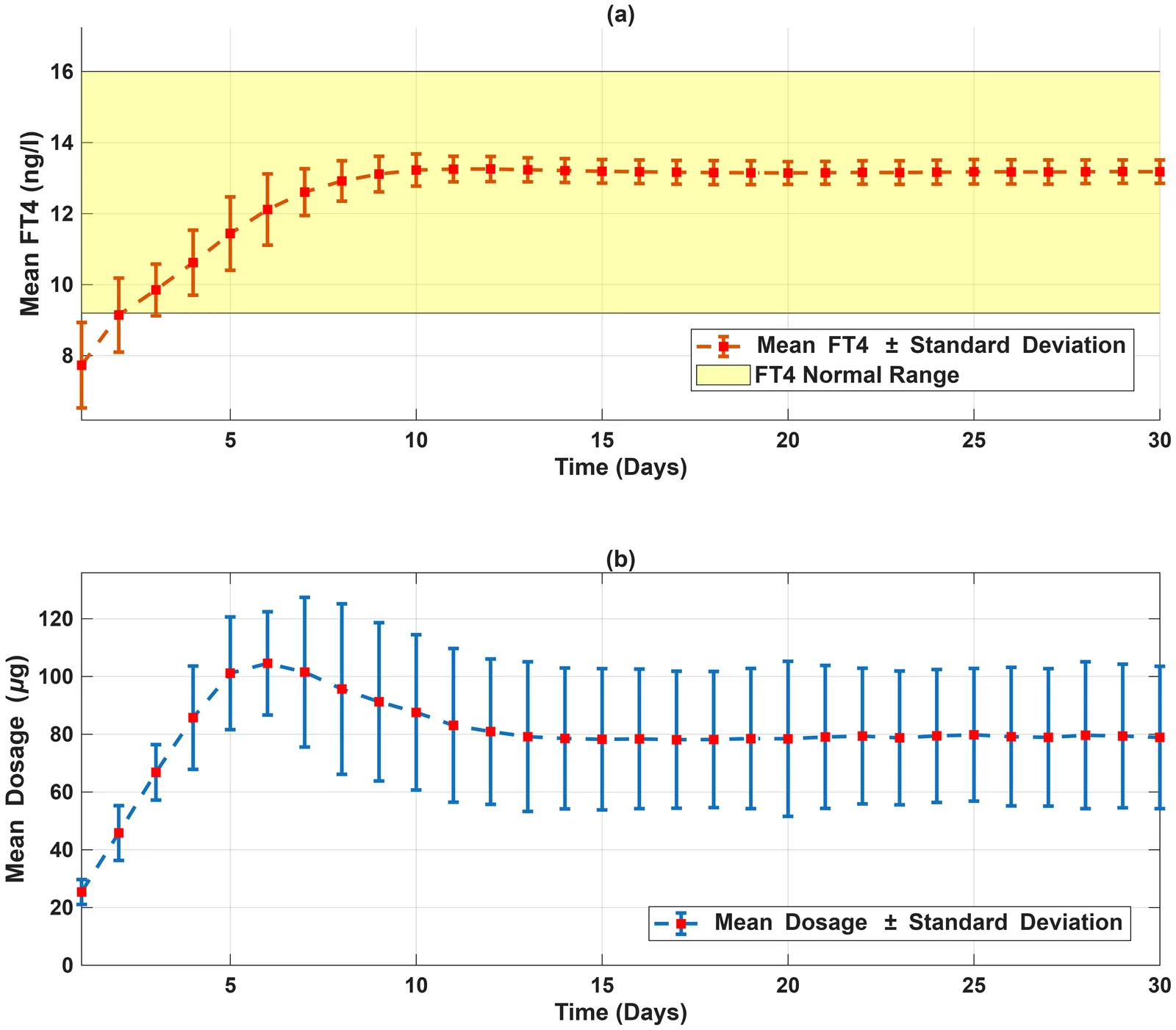
Population-level simulation results of the proposed ISS-LSTM-MPC-based LT4 dosing strategy for 50 heterogeneous simulated patients over a 30-day simulation period. **(a)** Mean and standard deviation of FT4 concentrations across the simulated population. **(b)** Corresponding LT4 dosage profiles across the simulated patients. At day 8, the mean FT4 concentration was 12.91 ± 0.56 ng/L, within the clinically accepted euthyroid range (9.2–16 ng/L).

The ISS-LSTM-MPC-based LT4 dosing strategy is applied to all simulated patients. **Figure 8a** shows the mean and standard deviation of FT4 concentrations, while **Figure 8b** presents the corresponding LT4 dosage profiles. The results indicate that, during the initial phase of treatment, the standard deviation in FT4 concentrations across patients is relatively high and generally below the euthyroid range (9.2–16 ng/L). As treatment progresses, the standard deviation decreases and FT4 concentrations converge toward the desired reference range, demonstrating consistent regulation across diverse patient conditions. At day 8, the mean FT4 concentration across the simulated population was 12.91 ± 0.56 ng/L, lying within the clinically accepted euthyroid range. In contrast, the standard deviation in the recommended LT4 dosage is initially low and increases during the later stages of treatment. This behavior reflects the ability of the proposed strategy to adjust dosing according to individual patient dynamics, resulting in personalized treatment responses.

Overall, these results demonstrate that the proposed ISS-LSTM-MPC-based dosing strategy achieves stable and personalized FT4 regulation across a heterogeneous patient population. This highlights the potential of the proposed framework for robust and individualized thyroid hormone therapy design.

## 4 Discussion

The present study developed and evaluated an ISS-LSTM-MPC-based LT4 dosing strategy for personalized thyroid hormone therapy in simulated patients with Hashimoto’s thyroiditis. The proposed framework successfully regulated FT4 concentrations toward patient-specific euthyroid levels while satisfying clinically relevant dosing constraints. Furthermore, robust performance was observed across simulated patients with varying biological parameters, demonstrating the capability of the proposed framework to maintain stable and individualized hormone regulation under heterogeneous patient conditions.

Accurate FT4 prediction despite variations in patient-specific biological parameters (**Figure 5**) demonstrates the robustness of the proposed LSTM-based prediction model under altered simulated patient dynamics. Although the LSTM network was trained using nominal parameter settings, it remained capable of accurately predicting FT4 concentrations when evaluated on simulated patients with modified biological parameters. This indicates that the developed data-driven model generalizes well across heterogeneous patient conditions and is suitable for control-oriented LT4 dosing applications.

The proposed ISS-LSTM-MPC framework successfully maintained FT4 concentrations within the patient-specific euthyroid range while satisfying clinically relevant dosing constraints. The recommended LT4 dosage profile respected the maximum admissible dosage variation of 25 *µ*g/day (**Figure 5a**), thereby avoiding abrupt hormonal changes. Correspondingly, the FT4 concentration generated by Thyrosim converged toward the personalized reference value of 13.1 ng/L after the reference update on day three and remained stable throughout the remainder of the 30-day simulation period (**Figure 5b**).

Reliable hormone regulation was also observed for a second simulated patient generated using further variations in Thyrosim’s biological parameters (**Figure 6**). The LSTM model continued to provide accurate FT4 predictions despite changing patient dynamics, further supporting the robustness of the proposed framework. In addition, the corresponding LT4 dosage profile satisfied all clinical dosing constraints, including limits on dosage magnitude and rate of change (**Figure 6a**). The FT4 concentration converged.

An additional simulation study was conducted for a simulated patient with an initial FT4 concentration of 4 ng/L (**Figure 7b**). The corresponding LT4 dosage profile (**Figure 7a**) demonstrates how the proposed controller adapts the daily LT4 dosage according to the patient’s individualized treatment requirements while satisfying the prescribed clinical dosing constraints. Despite the lower initial FT4 concentration, the proposed ISS-LSTM-MPC-based LT4 dosing strategy successfully regulated the FT4 concentration toward the personalized euthyroid reference within two weeks. This additional simulation further illustrates the applicability of the proposed framework across simulated patients exhibiting different initial thyroid hormone concentrations. These findings demonstrate the capability of the proposed framework to achieve stable and individualized hormone regulation across different simulated patient conditions.

Robustness across a heterogeneous patient population was further evaluated using 50 simulated patients generated by varying approximately 60 biological parameters of the Thyrosim model within their physiological ranges (**Figure 8**). During the initial treatment phase, the standard deviation of FT4 concentrations across simulated patients was relatively high and remained below the euthyroid range. As treatment progressed, the standard deviation decreased and FT4 concentrations converged toward the desired reference range, indicating consistent hormone regulation despite heterogeneous patient dynamics. In contrast, the variability in recommended LT4 dosages increased during later treatment stages, reflecting the controller’s ability to individualize therapy according to patient specific biological characteristics. Overall, these results demonstrate that the proposed ISS-LSTM-MPC framework achieves both robustness and personalization in LT4 therapy design.

From a control-theoretic perspective, the incorporation of ISS-based constraints ensures boundedness of the internal model states and contributes to closed-loop stability. Clinically, this translates to avoiding excessive hormone fluctuations while maintaining the patient within a safe and effective therapeutic range.

From a clinical perspective, the proposed framework may assist physicians in determining patient-specific LT4 dosages more efficiently, potentially reducing the need for frequent dose adjustments and clinical visits during the initial treatment phase.

Overall, the proposed ISS-LSTM-MPC-based LT4 dosing strategy represents a promising approach for personalized thyroid hormone therapy. By combining data-driven prediction with constraint-based optimal control, the framework achieves stable, robust, and individualized hormone regulation under varying simulated patient conditions. However, the current study is limited to in-silico validation using Thyrosim, and the use of simulated data may not fully capture real-world patient variability. Another practical limitation is the availability of sufficiently large and representative clinical datasets for training and validating machine learning models. Although the proposed hybrid framework utilizes physiologically meaningful simulation data generated by Thyrosim to train the LSTM model, future studies should investigate the integration of larger clinical datasets to further improve personalization, robustness, and clinical applicability. In addition, practical clinical considerations must be accounted for in future implementations. For example, LT4 medication is generally not administered before thyroid function testing, as recent LT4 intake may influence measured hormone concentrations and affect interpretation of thyroid status, highlighted in Pilz et al. (7).

While stability is enforced through ISS-based constraints, a formal analysis of recursive feasibility is not provided and remains an important topic for future investigation. Future studies should therefore focus on validation using clinical datasets, incorporation of measurement noise and physiological uncertainties, and assessment of real-world implementation feasibility. Moreover, future extensions of the proposed framework may incorporate physiological monitoring signals, such as heart-rate dynamics, to improve detection of LT4 overtreatment and further enhance individualized thyroid hormone therapy, see Pilz et al. (7).

## 5 Conclusion

This study presented a stability-aware ISS-LSTM-MPC framework for personalized LT4 dosing in simulated patients with Hashimoto’s thyroiditis. By combining data-driven FT4 prediction with constraint-based optimal control, the proposed approach achieved stable and individualized thyroid hormone regulation while satisfying clinically relevant dosing requirements. Robust performance across heterogeneous simulated patients further demonstrated the potential of the framework for personalized hormone therapy design. Although the current study is limited to in-silico evaluation using Thyrosim, the proposed methodology provides a promising foundation for future computer-assisted thyroid hormone therapy. Further work should focus on validation using clinical datasets, incorporation of measurement uncertainties, and assessment of real-world implementation feasibility.

## Data Availability

The raw data supporting the conclusions of this article will be made available by the authors, without undue reservation.

## Footnotes

1
http://biocyb1.cs.ucla.edu/thyrosim/cgi-bin/Thyrosim.cgi
