# Supplementary material for: Stability-aware LSTM–MPC framework for personalized levothyroxine dosing in Hashimoto’s thyroiditis
Source: Front Endocrinol (Lausanne). 2026 Aug 13;17:1894583. doi: 10.3389/fendo.2026.1894583 (PMC13518138; doi:10.3389/fendo.2026.1894583)
Supplement: Supplementary file 1 [file Supplementaryfile1.pdf]

Supplementary Material

S1 NOTATION

For a vector $a \in \mathbb{R}^n$, we denote its Euclidean norm by $|a|_2$, its 1-norm by $|a|_1$, and its transpose by a^T. The i-th component of a is denoted by a_i. For a sequence of vectors $\{a_k\}_{k\in\mathbb{N}} = \{a(0), a(1), \dots\}$, where each $a(k) \in \mathbb{R}^n$, we define the infinity norm of the sequence as

$$|\{a_k\}|_\infty = \sup_k\{|a(0)|_2, |a(1)|_2, |a(2)|_2, \dots, |a(k)|_2\}.$$

Given a real matrix B, we denote its induced norm by $|B|_p$, where $p = 1, 2, \infty$. At each discrete time step k, we write $x = x(k)$ for notational simplicity and denote the value at the subsequent time step by $x^+ = x(k+1)$. The Hadamard product between two vectors a and v is denoted by $a \circ v$. The sigmoid and hyperbolic tangent activation functions are expressed as

$$\sigma_g(x) = \frac{1}{1+e^{-x}} \quad \text{and} \quad \sigma_c(x) = \tanh(x),$$

where the operations are applied component-wise to each element of the vector $x \in \mathbb{R}^n$.

Later on it is exploited that these functions are bounded, i.e.,

$$\sigma_g(t) \in (0,1) \quad \forall t \in \mathbb{R}, \tag{S1}$$

$$\sigma_c(t) \in (-1,1) \quad \forall t \in \mathbb{R}, \tag{S2}$$

and Lipschitz continuous, with Lipschitz constants $L_g = 0.25$ and $L_c = 1$, respectively, as described in Bonassi et al. (2020). The matrices I_{N_P} and I_{N_c} are denoted as identity matrices of dimensions $N_P \times N_P$ and $N_c \times N_c$, respectively.

S2 LSTM NEURAL NETWORK ARCHITECTURE

Consider a single-layer LSTM neural network described by the original equations in Gers and Schmidhuber (2001), and reformulated to be strictly proper as in Bonassi et al. (2020). A strictly proper system is one in which the output does not depend directly on the current input, but only on the internal states. This structural constraint eliminates direct feed-through, ensuring a causal, delay-respecting input–output behavior. The system dynamics of the strictly proper LSTM network is given by:

$$h^+ = \sigma_g(W_f u + U_f \xi + b_f) \circ h + \sigma_g(W_i u + U_i \xi + b_i) \circ \sigma_c(W_c u + U_c \xi + b_c), \tag{S3a}$$

$$\xi^+ = \sigma_g(W_o u + U_o \xi + b_o) \circ \sigma_c(h^+), \tag{S3b}$$

$$y = C\xi + b_y. \tag{S3c}$$

where, $u \in \mathbb{R}^{n_u}$ is the network input, $h \in \mathbb{R}^{n_x}$ are the hidden states of the network, $\xi \in \mathbb{R}^{n_x}$ is known as the cell state of network, and $y \in \mathbb{R}^{n_y}$ is the prediction output of the network. The tunning parameters, are the the so-called weight matrices $W_f, W_i, W_c, W_o \in \mathbb{R}^{n_x \times n_u}$, $C \in \mathbb{R}^{n_y \times n_x}$, $U_f, U_i, U_c, U_o \in \mathbb{R}^{n_x \times n_x}$ and the so-called bias vectors b_f, b_i, b_c, b_o, b_y are selected of proper dimensions.

In this study, the input u is assumed to lie within a compact hypercube $\mathcal{U} = [-1, 1]^{n_u}$, reflecting the theoretical requirements for bounded input spaces,

$$u \in \mathcal{U} = [-1, 1]^{n_u}. \tag{S4}$$

Similarly, the cell state ξ is assumed to evolve within the open set $\Xi = (-1, 1)^{n_x}$, consistent with the saturation limits of the LSTM activation functions and as required for stability analysis derived in Bonassi et al. (2020),

$$\xi \in \Xi = (-1, 1)^{n_x}, \tag{S5}$$

and the bias vectors b_c are constant to the network (S3) and required to be identified during the training of the network. Equation (S3) could possibly re-write in standard form of a discrete-time, invariant, and nonlinear dynamical system, with $\chi = [h^T \quad \xi^T]$:

$$\chi^+ = f_{\text{LSTM}}(\chi, u, b_c), \tag{S6a}$$
$$y = g_{\text{LSTM}}(\chi). \tag{S6b}$$

S3 SPECIFIC CONDITIONS FOR ISS

In their study, Bonassi et al. (2020) introduce a framework for analyzing the ISS of LSTM neural networks, modelled through a set of gate functions (S7) and update equations (S3) that represents the network's dynamic behavior (see Figure S1).

$$f(u, \xi) = W_f u + U_f \xi + b_f, \tag{S7a}$$
$$i(u, \xi) = W_i u + U_i \xi + b_i, \tag{S7b}$$
$$c(u, \xi) = W_c u + U_c \xi + b_c, \tag{S7c}$$
$$o(u, \xi) = W_o u + U_o \xi + b_o, \tag{S7d}$$

where f, i, c and o are denoted as forget gate, input gate, candidate gate and output gate, respectively. Thus, state equations (S3) can be written as :

$$h^+ = \sigma_g(f(u, \xi)) \circ h + \sigma_g(i(u, \xi)) \circ \sigma_c(c(u, \xi)), \tag{S8a}$$
$$\xi^+ = \sigma_g(o(u, \xi)) \circ \sigma_c(h^+). \tag{S8b}$$

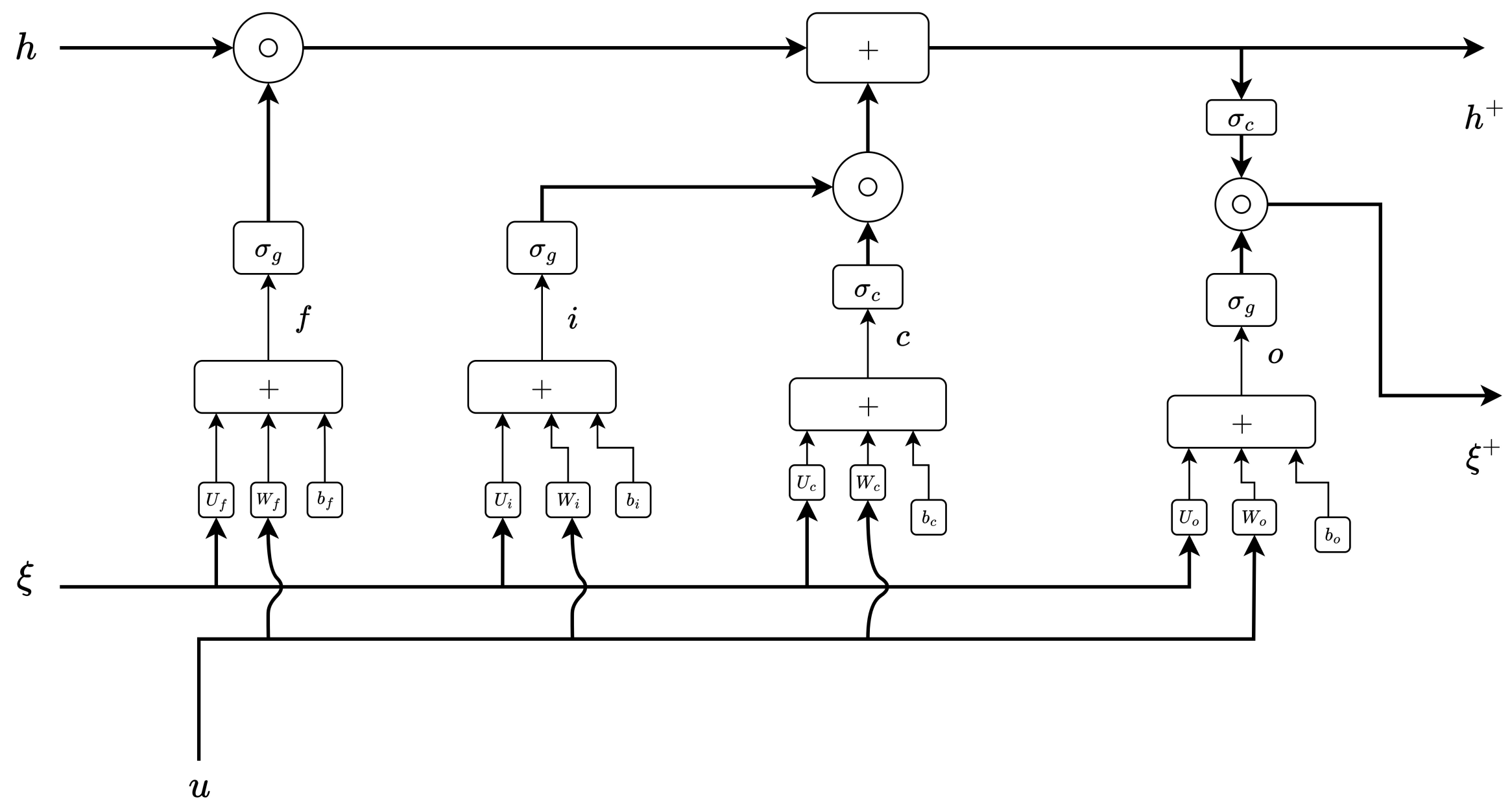

Figure S1. Structure of the LSTM cell as described by equations (S7)–(S8). The network receives the current input u, the current hidden state h, and cell state ξ. The network computes the forget gate $f(u, \xi)$, input gate $i(u, \xi)$, candidate gate $c(u, \xi)$, and output gate $o(u, \xi)$. These gates determine the updated hidden state h^+ and cell state ξ^+ by means of nonlinear operations σ_g and σ_c.

A possible candidate of ISS-Lyapunov function V, as defined in Bonassi et al. (2020) is given by

$$V(\chi) = |\chi|_1 = |h|_1 + |\xi|_1, \tag{S9}$$

were condition (S13) is satisfied with the functions

$$\psi_1(|\chi|_2) = |\chi|_2, \tag{S10a}$$

$$\psi_2(|\chi|_2) = \sqrt{2n_x}|\chi|_2. \tag{S10b}$$

Furthermore, as defined in Bonassi et al. (2020) a positive scalar δ should exist such that

$$\begin{aligned} (1 + |\alpha_o(u, \xi)|_1)|\alpha_f(u, \xi)|_1 - 1 \leq -\delta, \\ (1 + |\alpha_o(u, \xi)|_1)|\alpha_i(u, \xi)|_1|U_c|_1 - 1 \leq -\delta \end{aligned} \tag{S11}$$

where,

$$\begin{aligned} |\alpha_o(u, \xi)|_1 &\leq \sigma_g \left(|[W_o \; U_o \; b_o]|_\infty\right), \\ |\alpha_i(u, \xi)|_1 &\leq \sigma_g \left(|[W_i \; U_i \; b_i]|_\infty\right), \\ |\alpha_f(u, \xi)|_1 &\leq \sigma_g \left(|[W_f \; U_f \; b_f]|_\infty\right). \end{aligned} \tag{S12}$$

In the context of the above-mentioned equations, if a discrete-time, time-invariant, and nonlinear dynamical system (S6) admits,

$$\psi_1(|\chi|_2) \leq V(\chi) \leq \psi_2(|\chi|_2), \tag{S13a}$$

$$V(f_{\text{LSTM}}(\chi, u, b_c)) - V(\chi) \leq -\psi(|\chi|_2) + \sigma_u(|u|_2) + \sigma_b(|b_c|_2). \tag{S13b}$$

with,

$$\begin{aligned} -\psi(|\chi|_2) &= -\delta|\chi|_2, \\ \sigma_u(|u|_2) &= 2|W_c|_1\sqrt{n_u}|u|_2, \\ \sigma_b(|b_c|_2) &= 2\sqrt{n_x}|b_c|_2, \end{aligned} \tag{S14}$$

where n_x and n_u are the dimensions of the network states, network input and W_c is the weight matrix, then the system (S6) is ISS as specified in Bonassi et al. (2020), ensuring that the states (i.e., hidden and cell states) of system (S6) remain bounded under the influence of bounded inputs.

S4 STABILITY ANALYSIS

This section establishes the closed-loop stability of the proposed ISS-LSTM-MPC-based LT4 dosing strategy. The proposed framework ensures stability by incorporating ISS conditions as constraints within the MPC optimization problem. The control input d_k is computed at each time step based on the current LSTM state χ_k, where $\chi_k \in \mathbb{R}^{2n_x}$ contains the LSTM hidden and cell states, $\chi_k = [h_k^T, \xi_k^T]$.

S4.1 Closed-loop dynamics

In the proposed closed-loop system for thyroid hormone regulation, the control input d_k (i.e., daily oral LT4 dosage) is computed by solving an optimization problem based on the LSTM internal state χ_k. This input is defined by MPC's feedback policy $\pi(\chi_k)$, such that

$$d_k = \pi(\chi_k); \quad \text{see equation (S15c).}$$

We define the combined network input vector as

$$u_k = [d_k,\, x_k],$$

where $x_k = [x_{1_k}, x_{2_k}]$ represents the daily mean of absorbable LT4 in the gut and the total plasma T4 concentration at time step k, respectively.

The internal states of the LSTM network evolve as:

$$\chi_{k+1} = f_{\text{LSTM}}(\chi_k, u_k, b_c). \tag{S15a}$$

The predicted FT4 concentration is given by,

$$FT4_k = g_{\text{LSTM}}(\chi_k). \tag{S15b}$$

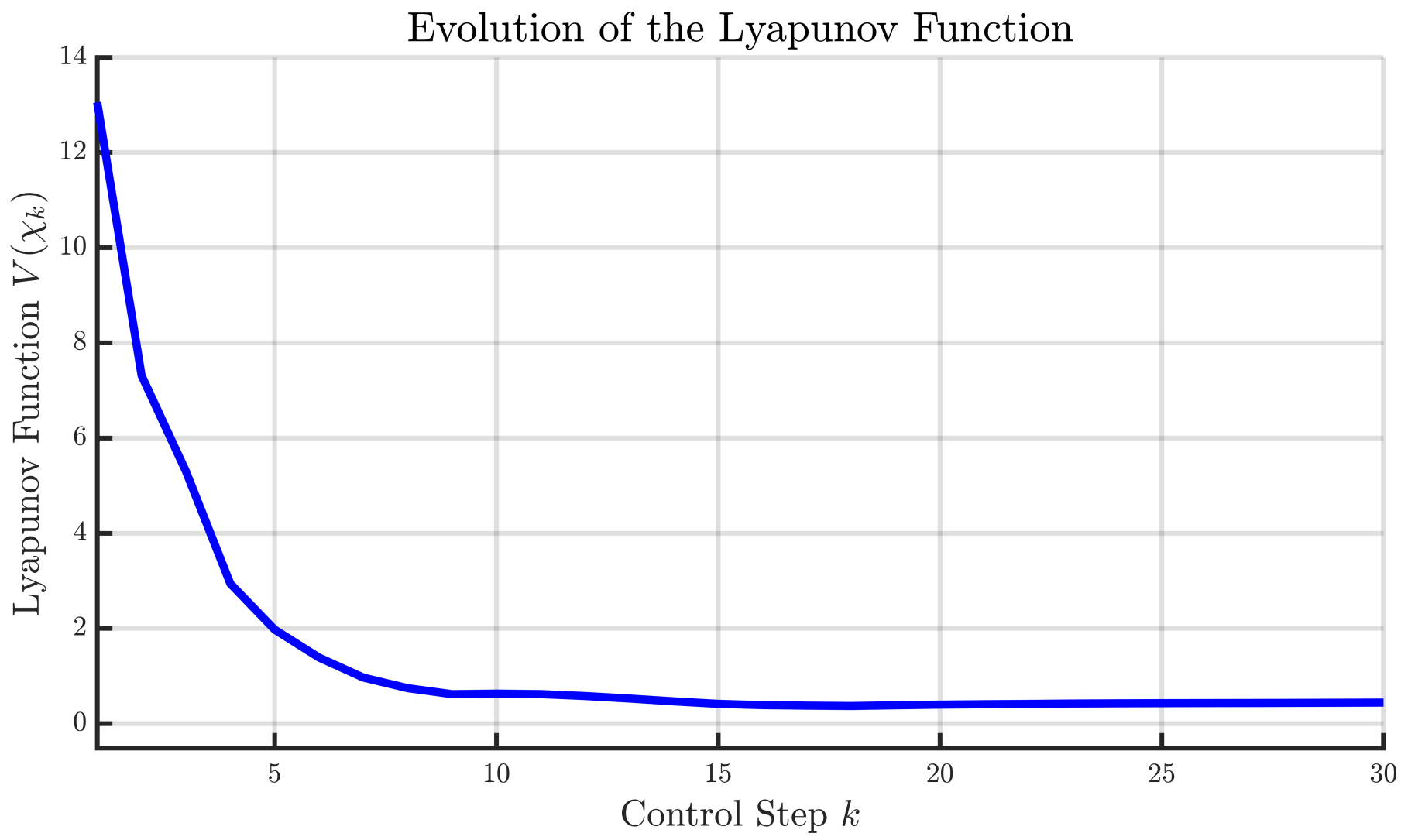

Figure S2. Evolution of the Lyapunov function V over 30 control steps during the closed-loop simulation of the ISS-LSTM-MPC framework. The Lyapunov function exhibits a sharp initial decrease, followed by convergence to a small positive value (approximately 0.4) with minor fluctuations.

At each time step k, the control policy is computed via,

$$\pi(\chi_k) = \arg\min_{d} J(\chi_k, d), \qquad \text{subject to constraints on } \chi_k \text{ and } d. \tag{S15c}$$

Over a prediction horizon N_p, the predicted LSTM state trajectory evolves as,

$$\chi_{k+i|k} = f_{\text{LSTM}}(\chi_{k+i-1|k}, u_{k+i-1}, b_c), \quad \text{where } u_{k+i-1} = [\pi(\chi_{k+i-1}),\, x_k], \quad i = 1, 2 \ldots, N_p. \tag{S15d}$$

Here, x_k is assumed to be fixed over the prediction horizon, consistent with the scenario where only the LSTM dynamics are used for prediction. The predicted FT4 concentration is given by,

$$FT4_{k+i|k} = g_{\text{LSTM}}(\chi_{k+i-1|k}),$$

based on the predicted LT4 dosage sequence $\{d_{k+i|k} = \pi(\chi_{k+i|k})\}_{i=0}^{N_c-1}$ and the fixed input x_k, utilizing the developed LSTM-based FT4 prediction model.

S4.2 Lyapunov-based stability condition

To ensure closed-loop stability under the proposed MPC control law, we define a Lyapunov function V and enforce the corresponding ISS conditions within the MPC optimization. The Lyapunov function V of the system described in (S15) is selected as,

$$V(\chi_k) = |\chi_k|_1. \tag{S16}$$

We adopt the ISS conditions from Bonassi et al. (2020), detailed in Section S3 :

$$\psi_1(|\chi_k|_2) \leq V(\chi_k) \leq \psi_2(|\chi_k|_2), \tag{S17a}$$

$$V(f_{\mathrm{LSTM}}(\chi_k, u_k, b_c)) - V(\chi_k) \leq -\psi(|\chi_k|_2) + \sigma_d(|u_k|_2) + \sigma_b(|b_c|_2), \tag{S17b}$$

where u_k is the network input vector, b_c is the LSTM bias, and $\psi_1, \psi_2, \psi, \sigma_d, \sigma_b$ are the functions described in Section S3. Evaluating $V(\chi_k)$ along the MPC-predicted trajectory and enforcing ISS conditions (S17) as constraints in the optimization problem ensures ISS of the overall closed-loop system.

Figure S2 illustrates the evolution of the Lyapunov function $V(\chi_k)$ over time, providing empirical evidence for the ISS of the closed-loop system. In the initial phase, $V(\chi_k)$ rapidly decreases from approximately 13.06 to below 1.3 within six time steps, indicating fast stabilization of the LSTM internal states under the optimized MPC policy. Subsequently, $V(\chi_k)$ remains bounded within the range 0.33 to 0.44, exhibiting non-vanishing, yet stable behavior. This trend is consistent with ISS theory in that the Lyapunov function V need not decay to zero in the presence of persistent inputs and modeling inaccuracies but must remain bounded. This behavior is formally explained by the ISS condition (S17b), which ensures a decrease in $V(\chi_k)$ whenever the state norm dominates the influence of the control input and model bias.

By enforcing these conditions (S17) as constraints within the MPC optimization and applying control in a receding horizon fashion, the closed-loop system inherits the ISS property. As demonstrated in Stiti et al. (2024), such recursive enforcement of Lyapunov constraints ensures closed-loop stability. In this study, we adapt this principle to an LSTM-based nonlinear system by employing analytically derived ISS conditions suitable for black-box neural dynamics. The results validate the effectiveness of the proposed ISS-LSTM-MPC framework in ensuring closed-loop stability despite the nonlinear and black-box nature of the model.

S4.2.0.1 Implication for stability

Since the control input $\pi(\chi_k)$ is computed as described in (S15c) and the predicted states $\chi_{k+i|k}$ evolve under this sequence that ensures ISS conditions (S17) are satisfied along the trajectory. Therefore, by enforcing ISS-based Lyapunov constraints at each time step, the proposed ISS-LSTM-MPC framework ensures closed-loop stability, even under nonlinear, black-box dynamics. A formal analysis of recursive feasibility under ISS constraints and black-box neural dynamics is left for future investigation, but the current results strongly indicate robustness of the proposed framework in simulation settings.

In clinical terms, enforcing Lyapunov-based ISS conditions ensures that thyroid hormone concentrations and LT4 adjustments remain bounded, thereby avoiding extreme fluctuations that could potentially harm individual metabolic stability.

S5 THYROSIM MODEL PARAMETERS USED FOR POPULATION GENERATION

Table S1: Nominal values and reported coefficients of variation (CVs) of the Thyrosim model parameters reproduced from Han et al. (2016). The original sources of the individual parameter values are listed in the *Source* column. During the robustness analysis, patient-specific physiological parameters with reported CVs were perturbed around their nominal values according to the corresponding CV to generate a heterogeneous population of simulated patients. Parameters without reported CVs were retained at their nominal values.

Parameter	Units	Estimate	%CV	Source
ϕ	h	-3.71	1.04	Eisenberg et al. (2008)
A_0	μmol/h	581	61.4	Eisenberg et al. (2008)
B_0	μmol/h	1166	60.7	Eisenberg et al. (2008)
$k_3 = k_4$	μmol/h	0.118	6.43	Eisenberg et al. (2008)
f_4 range	h^{-1}	0.118–0.708	–	Eisenberg et al. (2010)
$k_{T_3B}^{deg}$	h^{-1}	0.037	12.6	Eisenberg et al. (2008)
V_P (kids)	L	1	–	Ben-Shachar et al. (2012)
V_P (adults)	L	3.2	–	Eisenberg et al. (2008)
V_{TSH} (kids)	L	2.5	–	Ben-Shachar et al. (2012)
V_{TSH} (adults)	L	5.2	–	Eisenberg et al. (2008)
$K_m^{D\,fast1}$	μmol	2.85	–	Eisenberg et al. (2008)
$K_m^{D\,slow1}$	μmol	95	–	Eisenberg et al. (2008)
$K_m^{D\,slow2}$	μmol	0.075	–	Eisenberg et al. (2008)
$v_{max}^{D\,fast1}$	μmol/h	9.99×10^{-3}	30.6	Eisenberg et al. (2008)
$v_{max}^{D\,slow1}$	μmol/h	6.63×10^{-4}	6.27	Eisenberg et al. (2008)
$v_{max}^{D\,slow2}$	μmol/h	7.46×10^{-4}	6.27	Eisenberg et al. (2008)
S_3	h^{-1}	3.36×10^{-4}	6.49	Eisenberg et al. (2008)
S_4	h^{-1}	1.74×10^{-3}	7.4	Eisenberg et al. (2008)
τ	h	8	–	Eisenberg et al. (2008)
k_4^{absorb}	h^{-1}	0.88	2.2	Eisenberg et al. (2008)
$k_4^{dissolve}$	h^{-1}	1.3	–	Eisenberg et al. (2008)
$k_4^{excrete}$	h^{-1}	0.12	16.3	Eisenberg et al. (2008)
k_3^{absorb}	h^{-1}	0.88	7.2	Eisenberg et al. (2008)
$k_3^{dissolve}$	h^{-1}	1.78	32.0	Eisenberg et al. (2008)
$k_3^{excrete}$	h^{-1}	0.12	7.2	Eisenberg et al. (2008)
A	unitless	0.000289	–	Eisenberg et al. (2006)
B	$mmol^{-1}$	0.000214	–	Eisenberg et al. (2006)
C	$mmol^{-2}$	0.000128	–	Eisenberg et al. (2006)
D	$mmol^{-3}$	-8.83×10^{-6}	–	Eisenberg et al. (2006)
a	unitless	0.00395	–	Eisenberg et al. (2006)
b	$mmol^{-1}$	0.00185	–	Eisenberg et al. (2006)
c	$mmol^{-2}$	0.000610	–	Eisenberg et al. (2006)
d	$mmol^{-3}$	-0.000505	–	Eisenberg et al. (2006)

Continued on next page

Table S1 – continued from previous page

Parameter	Units	Estimate	%CV	Source
k_{02} (kids)	h^{-1}	0.0114	17.0	Ben-Shachar et al. (2012)
k_{12} (kids)	h^{-1}	0.523	19.2	Ben-Shachar et al. (2012)
k_{13} (kids)	h^{-1}	0.0514	28.8	Ben-Shachar et al. (2012)
k_{21}^{free} (kids)	h^{-1}	2275	14.4	Ben-Shachar et al. (2012)
k_{31}^{free} (kids)	h^{-1}	255	36.0	Ben-Shachar et al. (2012)
k_{02} (adults)	h^{-1}	0.0189	25.7	Eisenberg et al. (2006)
k_{12} (adults)	h^{-1}	0.868	18.3	Eisenberg et al. (2006)
k_{13} (adults)	h^{-1}	0.108	12.4	Eisenberg et al. (2006)
k_{21}^{free} (adults)	h^{-1}	1503	31.2	Eisenberg et al. (2006)
k_{31}^{free} (adults)	h^{-1}	584	16.6	Eisenberg et al. (2006)
k_{54}^{free}	h^{-1}	2043	–	Eisenberg et al. (2006)
k_{64}^{free}	h^{-1}	127	–	Eisenberg et al. (2006)
k_{05}	h^{-1}	0.207	12.8	Eisenberg et al. (2006)
k_{45}	h^{-1}	5.37	16.3	Eisenberg et al. (2006)
k_{46}	h^{-1}	0.0689	4.79	Eisenberg et al. (2006)
A_{max}	mmol/h	2.37	61.4	Eisenberg et al. (2010)
$k_{deg,TSH}^{HYPO}$	h^{-1}	0.53	–	Eisenberg et al. (2010)
K_{LAG} (kids)	unitless	6.5	–	Ben-Shachar et al. (2012)
K_{LAG} (adults)	unitless	5	–	Eisenberg et al. (2010)
k_{LAG}^{HYPO}	h^{-1}	0.0034	5.87	Eisenberg et al. (2010)
K_{50}^{TSH}	μmol	23	–	Eisenberg et al. (2010)
T_{3P}^{EU} (kids)	nmol/L	2.6	–	Han et al. (2016)
T_{4P}^{EU} (kids)	nmol/L	100.8	–	Han et al. (2016)
T_{3P}^{EU} (adults)	μmol	0.006	–	Eisenberg et al. (2010)
T_{4P}^{EU} (adults)	μmol	0.29	–	Eisenberg et al. (2010)
V_{max}^{TSH}	h^{-1}	0.037	12.6	Eisenberg et al. (2010)
Parameters for the linearized model				
k_{21}	h^{-1}	0.544	31.2	Eisenberg et al. (2006)
k_{31}	h^{-1}	0.211	16.6	Eisenberg et al. (2006)
k_{54}	h^{-1}	9.24	13.7	Eisenberg et al. (2006)
k_{64}	h^{-1}	0.573	6.20	Eisenberg et al. (2006)

REFERENCES

Bonassi F, Terzi E, Farina M, Scattolini R. Lstm neural networks: Input to state stability and probabilistic safety verification. *Proceedings of the 2nd Conference on Learning for Dynamics and Control* (PMLR) (2020), *Proceedings of Machine Learning Research*, vol. 120, 85–94.

Gers F, Schmidhuber E. Lstm recurrent networks learn simple context-free and context-sensitive languages. *IEEE Transactions on Neural Networks* **12** (2001) 1333–1340. doi:10.1109/72.963769.

Stiti C, Benrabah M, Aouaichia A, Oubelaid A, Bajaj M, Tuka MB, et al. Lyapunov-based neural network model predictive control using metaheuristic optimization approach. *Scientific Reports* **14** (2024). doi:10.1038/s41598-024-69365-9.

Han SX, Eisenberg M, Larsen PR, DiStefano J. Thyrosim app for education and research predicts potential health risks of over-the-counter thyroid supplements. *Thyroid* **26** (2016) 489–498. doi:10.1089/thy.2015.0373.

Eisenberg M, Samuels M, DiStefano JJ. Extensions, validation, and clinical applications of a feedback control system simulator of the hypothalamo-pituitary-thyroid axis. *Thyroid* **18** (2008) 1071–1085. doi:10.1089/thy.2007.0388.

Eisenberg MC, Santini F, Marsili A, Pinchera A, DiStefano JJ. Tsh regulation dynamics in central and extreme primary hypothyroidism. *Thyroid* **20** (2010) 1215–1228. doi:10.1089/thy.2009.0349.

Ben-Shachar R, Eisenberg MC, Huang SA, DiStefano III JJ. Simulation of post-thyroidectomy treatment alternatives for triiodothyronine or thyroxine replacement in pediatric thyroid cancer patients. *Thyroid* **22** (2012) 595–603. doi:10.1089/thy.2011.0355.

Eisenberg MC, Samuels MH, DiStefano III JJ. L-t4 bioequivalence and hormone replacement studies via feedback control simulations. *Thyroid* **16** (2006) 1279–1292. doi:10.1089/thy.2006.0144.
